# Convergence in Maximum Stomatal Conductance of C_3_ Woody Angiosperms in Natural Ecosystems Across Bioclimatic Zones

**DOI:** 10.3389/fpls.2019.00558

**Published:** 2019-05-07

**Authors:** Michelle Murray, Wuu Kuang Soh, Charilaos Yiotis, Sven Batke, Andrew C. Parnell, Robert A. Spicer, Tracy Lawson, Rodrigo Caballero, Ian J. Wright, Conor Purcell, Jennifer C. McElwain

**Affiliations:** ^1^Department of Botany, School of Natural Sciences, Trinity College Dublin, Dublin, Ireland; ^2^Department of Biology, Edge Hill University, Ormskirk, United Kingdom; ^3^Hamilton Institute, Maynooth University, Maynooth, Ireland; ^4^Xishuangbanna Tropical Botanical Garden, Chinese Academy of Sciences, Menglun, China; ^5^School of Environment, Earth and Ecosystem Sciences, The Open University, Milton Keynes, United Kingdom; ^6^School of Biological Sciences, University of Essex, Colchester, United Kingdom; ^7^Department of Meteorology, Stockholm University, Stockholm, Sweden; ^8^Department of Biological Sciences, Faculty of Science, Macquarie University, Sydney, NSW, Australia

**Keywords:** biomes, convergence, habitat, maximum stomatal conductance, natural ecosystems, understory, variance, woody angiosperms

## Abstract

Stomatal conductance (*g*_s_) in terrestrial vegetation regulates the uptake of atmospheric carbon dioxide for photosynthesis and water loss through transpiration, closely linking the biosphere and atmosphere and influencing climate. Yet, the range and pattern of *g*_s_ in plants from natural ecosystems across broad geographic, climatic, and taxonomic ranges remains poorly quantified. Furthermore, attempts to characterize *g*_s_ on such scales have predominantly relied upon meta-analyses compiling data from many different studies. This approach may be inherently problematic as it combines data collected using unstandardized protocols, sometimes over decadal time spans, and from different habitat groups. Using a standardized protocol, we measured leaf-level *g*_s_ using porometry in 218 C_3_ woody angiosperm species in natural ecosystems representing seven bioclimatic zones. The resulting dataset of 4273 *g*_s_ measurements, which we call STraits (Stomatal Traits), was used to determine patterns in maximum *g*_s_ (*g*_smax_) across bioclimatic zones and whether there was similarity in the mean *g*_smax_ of C3 woody angiosperms across ecosystem types. We also tested for differential *g*_smax_ in two broadly defined habitat groups – open-canopy and understory-subcanopy – within and across bioclimatic zones. We found strong convergence in mean *g*_smax_ of C3 woody angiosperms in the understory-subcanopy habitats across six bioclimatic zones, but not in open-canopy habitats. Mean *g*_smax_ in open-canopy habitats (266 ± 100 mmol m^-2^ s^-1^) was significantly higher than in understory-subcanopy habitats (233 ± 86 mmol m^-2^ s^-1^). There was also a central tendency in the overall dataset to operate toward a *g*_smax_ of ∼250 mmol m^-2^ s^-1^. We suggest that the observed convergence in mean *g*_smax_ of C3 woody angiosperms in the understory-subcanopy is due to a buffering of *g*_smax_ against macroclimate effects which will lead to differential response of C3 woody angiosperm vegetation in these two habitats to future global change. Therefore, it will be important for future studies of *g*_smax_ to categorize vegetation according to habitat group.

## Introduction

Biosphere–atmosphere processes are intrinsically linked and the functioning of land plants is a critical component (Cox et al., 1998; Hutjes et al., 1998). Understanding the plant–atmosphere interface informs our ability to describe, understand and predict the Earth system. Through photosynthesis and transpiration, plants couple the carbon and water cycles and thereby play a pivotal role in Earth system and plant-climate feedbacks (Hetherington and Woodward, 2003; Keenan et al., 2014; Schlesinger and Jasechko, 2014; Lin et al., 2015). These plant physiological processes are influenced, either directly or indirectly, by many biotic (competition/interaction) and abiotic (light, temperature, nutrient, and water requirements) factors, and by biochemical and physical pathways which regulate the exchanges of gasses in these processes, such as stomatal conductance (*g*_s_).

Stomata are minute valves on the plant leaf surface consisting of two turgor-operated guard cells surrounding a central pore. In response to fluctuating external signals (light, temperature and humidity, soil moisture, and nutrient status) and also internal signals (guard cell and mesophyll), the apertures of stomata are adjusted to regulate the trade-off between CO_2_ uptake for photosynthesis and the inevitable loss of water via transpiration (Farquhar and Sharkey, 1982; Schulze et al., 1994; Hutjes et al., 1998; Hetherington and Woodward, 2003; Mott, 2009; Franks et al., 2013; Lawson and Blatt, 2014; McAusland et al., 2016). Stomatal developmental traits (e.g., number and size) are also modified in response to external stimuli such as elevated atmospheric CO_2_ (Woodward, 1987; Woodward and Kelly, 1995; Haworth et al., 2013) which, in turn, sets the maximum theoretical limits for stomatal conductance (Franks and Beerling, 2009; Dow et al., 2014; Franks et al., 2014; McElwain et al., 2016) within the phenotypic range of each species.

A significant body of research has been published on stomatal responses to atmospheric change in woody vegetation (Schulze et al., 1994; Körner, 1995; Medlyn et al., 2001; Ainsworth and Rogers, 2007; Leuzinger and Körner, 2007; Keenan et al., 2013; Schlesinger and Jasechko, 2014) and on the implications of such responses for climate (Gedney et al., 2006; Betts et al., 2007), climate modeling (Medlyn et al., 2011; Frank et al., 2015) and reconstructing past atmospheric CO_2_ concentration (Franks et al., 2014; McElwain and Steinthorsdottir, 2017; McElwain, 2018). Previous meta-analyses have resulted in compilations of important global *g*_s_ datasets, in which portions of the data were contributed to by the authors themselves (Körner, 1995; Lin et al., 2015; Maire et al., 2015). While these datasets provide values for maximum *g*_s_ from global biomes, the characterization of range and pattern in field-measured *g*_s_ across diverse bioclimatic zones using such datasets is problematic for three reasons. Firstly, the use of many different protocols from different studies may affect *g*_s_ values. Secondly, due to temporal differences between studies collected over decades, *g*_s_ data may have been collected under different atmospheric CO_2_ concentrations. Rising atmospheric CO_2_ is known to cause an increase in CO_2_ concentration in the leaf mesophyll, triggering responses in the guard cells and mediating stomatal movement in the short-term, and to down-regulate development of stomata in the long term, thus affecting stomatal conductance (Engineer et al., 2016). Thirdly, differential *g*_s_ of plants from different habitats (e.g., open versus shaded habitats) may not have been considered in the different studies. Currently, there are concerns around using such datasets, in which data from lower canopy, “shade” leaves is mixed in unknown proportions with that from outer canopy, “sun” leaves (Keenan and Niinemets, 2016). For these reasons, meaningful comparison of *g*_s_ data from published datasets is difficult to achieve.

To compare the stomatal conductance of vegetation from different bioclimatic zones it is important to firstly standardize a data collection method. It may also be important that data have been collected within narrow time spans, to minimize the influence of rapidly rising atmospheric CO_2_ on *g*_s_. Additionally, as *g*_s_ is affected by environmental conditions, it is potentially important to distinguish plants based on common environmental niches, for instance, open habitats versus more shaded habitats, since differences in microclimatic variables such as light, wind speed, temperature and relative humidity in different habitats can affect stomatal response. Indeed, light plays a large part in the stomatal development of developing leaves through the sensing by mature leaves of shifting light availability (Casson and Gray, 2008). Leaf-level stomatal conductance is also affected by the static boundary layer around the leaf. Boundary layer resistance may be higher in understory plant leaves because of the attenuation of wind speed by the surrounding canopy (Jarvis and McNaughton, 1986; Daudet et al., 1999), which may result in a general decrease in stomatal conductance in such environments. To date, tests on the differential stomatal conductance of vegetation in different habitats have not been undertaken on a large geographic and taxonomic scale. A comparison of leaf diffusive conductances among major global vegetation types by Körner (1995) reported little difference in the maximum field stomatal conductance of woody species between the major world biomes; however, this pattern was not formally statistically tested.

In this study we explored patterns in *in situ* maximum stomatal conductance (*g*_smax_) of C_3_ woody angiosperms in their natural field environment, across broad geographic, climatic and taxonomic ranges. By maximum stomatal conductance we mean the highest conductance on fully expanded leaves, measured during the summer growing season. First, we investigated whether there was similarity or convergence in *g*_smax_ of C_3_ woody angiosperms in two broadly defined habitat groups, namely open-canopy and understory-subcanopy (by convergence in *g*_smax_ we mean no significant difference in mean *g*_smax_ of C3 woody angiosperms between bioclimatic zones and/or across habitats). Secondly, we investigated whether there was a common central tendency in *g*_smax_ between this study and a published meta-analysis (Maire et al., 2015). To address these questions, we used a new dataset of field-measured *g*_s_, called STraits (Stomatal Traits), which we collected using the same porometry-based protocol across multiple species and bioclimatic zones over a period of 3 years (2012–2015), a period over which CO_2_ has risen by less than ∼7 ppm.

## Materials and Methods

### Site Selection and Bioclimatic ZoneIdentification

This study forms part of a larger, ongoing project comparing the stomatal traits of historical and present-day woody angiosperm leaves in response to rising atmospheric CO_2_. The reference point for that project was a unique geo-referenced collection of woody dicot leaf physiognomic data representing the major global biomes, known as the Climate-Leaf Analysis Multivariate Program (CLAMP) (Wolfe, 1993; Yang et al., 2015), housed at the Smithsonian National Museum of Natural History (NMNH), Washington, DC, United States. For this current study, we chose our sites from a subset of the CLAMP database dating from the late 1980s (Wolfe, 1993), which provided a broad collection of voucher herbarium specimens from sites located close to meteorological stations. The aim was to assemble a dataset of stomatal conductance measurements capturing the *in situ* leaf stomatal conductance variability in a wide range of C_3_ woody angiosperm species at the levels of habitat and bioclimatic zone, across many bioclimatic zones. In this study, bioclimatic zone refers to broad regions of vegetation type at different latitudinal gradients, adapted from Whittaker’s classification of vegetation biomes according to mean annual precipitation and mean annual temperature (Whittaker, 1975) (Table 1).

**Table 1:** Sampling site information summary arranged according to latitude.

Bioclimatic zone	State	Site	Collection date	Lat./long.	Elevation (m asl)	Vegetation composition	Soil/Geology	MAT (°C)	MAP (mm)
Boreal forest	Alaska	Eklutna Lake, Chugach SP	August 2014	61°25′N, 149°09′W	870–900	Mixed coniferous and broadleaved deciduous	Soil deep alluvial to shallow, rocky, metamorphic-derived	–1.44	48.07
Boreal forest	Alaska	Bird Creek, Chugach SP	August 2014	60°57′N, 149°06′W	70–100	Mixed coniferous-broadleaved deciduous	Soil deep to shallow, rocky, metamorphic-derived	0.36	48.09
Boreal forest	Alaska	Captain Cook SRA	August 2014	60°33′N, 151°12′W	18	Mixed coniferous and broadleaved deciduous	Soil deep, sedimentary-derived and glacial outwash	3.20	53.21
Temperate rainforest	Oregon	Bandon SP	August 2013	43°07′N, 124°23′W	2–30	Coastal strand, bog/dunes; coniferous forest; roadside	Soil sandy	12.20	119.80
Temperate rainforest	Oregon	Cape Blanco SP	August 2013	42°50′N, 124°32′W	1–65	Coastal headland; coniferous forest; freshwater wetland; riparian shrub	On bluffs, soil sandy; on banks of Sixes River soil deep alluvial	11.18	85.45
Temperate rainforest	Oregon	Port Orford SP	August 2013	42°45′N, 124°30′W	10	Coastal headland; coastal strand, dunes; coniferous forest	On flats around Garrison Lake, soil sandy; on slopes and on alluvial flats, soil deep alluvial to sedimentary-derived	11.18	85.45
Temperate deciduous forest	Pennsylvania	Gouldsboro SP (Mount Pocono)	May 2013	41°14′N, 75°28′W	580–600	Mixed deciduous: American beech, oak, red maple	Soil deep, derived from sedimentary rocks	9.05	97.70
Temperate deciduous forest	Pennsylvania	Big Pocono SP (Stroudsburg)	May 2013	40°58′N, 75°11′W	600–640	Barrens, scrub/mixed oak	Soil deep, derived from sedimentary rocks	9.05	97.70
Temperate deciduous forest	Maryland	SERC	June 2014	38°52′N, 76°35′W	30–50	Oak-hickory forest, tulip-popular, red maple	Soil deep, derived from sedimentary rocks	14.22	94.82
Temperate deciduous forest	Maryland	Battle Creek Cypress Swamp	May 2013	38°29′N, 76°35′W	<10	Closed-canopy *Taxodium* swamp	Mostly swamp soil; in closed-canopy deciduous forest, soil deep, derived from sedimentary rocks	14.50	66.95
Mediterranean	California	Half Moon Bay	June 2013	37°25′N, 122°26′W	10–20	Coastal bluff scrubs; coastal dunes/riparian	Soil deep alluvial	14.55	144.46
Mediterranean	California	Jasper Ridge Biological Preserve	June 2013	37°24′N, 122°14′W	30–50	Chamise chaparral; coast live oak; riparian scrub	Soil deep alluvial along creeks to shallow, rocky serpentine-derived on slopes	13.41	52.08
Mediterranean	California	New Brighton Beach SP	July 2013	36°58′N, 121°55′W	2–40	Coast range mixed coniferous; coastal bluff scrub	Soil deep alluvial	14.32	44.88
Subtropical desert	Arizona	Punkin Center, Tonto NF	July 2013	33°52′N, 111°20′W	710–780	Sonoran Desert riparian shrub	Along streambeds, soil shallow, rocky, derived from metamorphic rocks; on flats above Tonto Creek, soil shallow alluvial; in open desert alluvial plain soil deep alluvial	17.83	97.86
Subtropical desert	Arizona	Roosevelt Lake, Tonto NF	July 2013	33°41.6′N, 111°10′W	645–695	Sonoran Desert riparian shrub	On slopes and ephemeral stream-beds soil shallow, rocky, derived from volcanic rocks	15.66	39.33
Subtropical desert	Arizona	Saguaro Lake, Tonto NF	July 2013	33°33′N, 111°32′W	470–490	Sonoran Desert riparian shrub	In and along ephemeral streambeds, soil derived from granite; open desert floodplain of Salt River soil deep alluvial	17.83	39.33
Tropical seasonal forest (moist)	Puerto Rico	Bosque Estatal de Guajataca	July 2014	18°24′N, 66°58′W	250	Subtropical moist forest	Karst topography, limestone-derived soil	24.15	176.54
Tropical rainforest	Fiji	Natua	June 2015	16°43′S, 179°09′E	50–70	Broadleaf evergreen tropical rainforest	Poorly drained sandy loam soils derived from strongly weathered andesite	24.58	168.86
Tropical rainforest	Fiji	Seaqaqa	May and June 2015	16°38′S, 179°09′E	90–200	Broadleaf evergreen tropical rainforest	Soil deep, well-drained, friable, and clay, derived from strongly weathered (red) andesite	24.58	168.86

*Mean annual temperature (MAT) and mean annual precipitation (MAP) are from Harris et al. (2013). Soil/geology information for each site taken from Wolfe (1993), except Fiji, which is taken from Laffan (1988). SP, State Park; NF, National Forest; SRA, State Recreation Area.*

**FIGURE 1:**
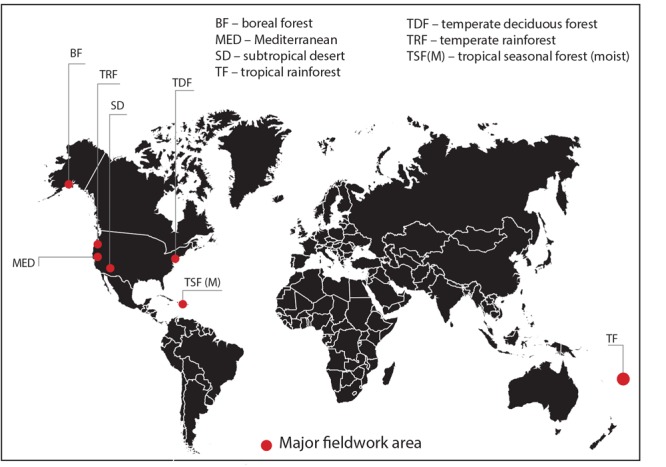
Location map of major study areas representing seven bioclimatic zones (see Table 1 for sampling and site information).

Nineteen sites were selected across seven bioclimatic zones at latitudes from 61°57′N to 16°43′S: boreal forest, temperate rainforest, temperate deciduous forest, Mediterranean woodland/shrubland, subtropical desert, tropical seasonal forest (moist) and tropical rainforest (Figure 1). Each bioclimatic zone was represented by between two and four sampling sites within not more than three degrees latitude of each other. The focus of this study was on the variability and patterns in stomatal conductance in C_3_ woody angiosperms only and we do not claim that results presented here are representative of all species or vegetation within those bioclimatic zones. However, our data certainly capture some of the *in situ* stomatal conductance variability at the level of species of a representative portion of the C_3_ woody angiosperm group of that bioclimatic zone. We have therefore retained most of the biome names from Whittaker (1975) to identify our bioclimatic zones and have used the abbreviations of those names where applicable. We restricted site selection to locations below 800 m above sea level to limit the influence of decreasing CO_2_ partial pressure (*p*CO_2_) on *g*_s_ at high altitudes (McElwain, 2004). All but two sites are in the northern hemisphere. The two tropical southern hemisphere sites experience a predominantly all-year-round growing season. Fieldwork was carried out during the growing season in each bioclimatic zone, that is, from May to August 2013, June to August 2014 and May to June 2015 (see Table 1 for fieldwork summary). None of the sites was experiencing drought stress at time of measurements. In the subtropical desert, measurements were taken during the monsoon season. In order to obtain a representative sample of C_3_ woody angiosperm species within the boreal forest bioclimatic zone, which is dominated by conifers, sampling was conducted within the interior boreal forest zone of Alaska, which has extensive areas of open and closed deciduous forests (Viereck et al., 1992).

### Species Selection

Only broadleaf C_3_ woody angiosperm species were sampled for this study (gymnosperms, grasses, and crops were not included). Species were selected for stomatal conductance measurement from the CLAMP collection of woody angiosperm leaves originally sampled between 1988 and 1991 by Wolfe (1993). This provided a target list of between 10 and 28 species per site, and between 18 and 56 species per bioclimatic zone. We identified any CLAMP leaves not already identified to species level using the relevant regional floras (Seemann, 1865–1873; Flora of North America Editorial Committee, 1993+) and taxonomic nomenclature was updated where necessary.

### Stomatal Conductance and Microclimate Measurements

Three to four individuals from each species were identified at each site and one leaf from each individual was tagged for *g*_s_ measurement. Fully expanded, healthy, pest- and pathogen-free leaves were chosen from well-lit regions of the canopy. Based on our field observations we determined two broadly defined habitat groups: open-canopy and understory-subcanopy. For this study, open-canopy refers to plants that are located either in open areas or at the forest canopy edge and receiving direct sunlight. By contrast, understory-subcanopy refers to plants occurring within the forest canopy, in shade but receiving sunflecks. Plants from the subtropical desert bioclimatic zone were all classified as open-canopy. In both habitat groups leaves were measured at heights reachable with a porometer, about 3 m or less. It is also known that *g*_s_ varies with tree height and this protocol standardizes all *g*_s_ measurements. A modification of the variance protocol of McElwain et al. (2016) was used to record the natural day-to-day variability in *g*_s_ for each species under ambient conditions, where *g*_s_ was measured on each leaf once per day at approximately the same time each day over 3–4 days.

We measured leaf diffusive conductance (to water) for this study, however, we have used the general term ‘stomatal conductance’ throughout this manuscript, as we assumed cuticular conductance to be negligible, therefore we took diffusive conductance to approximate *g*_s_. All stomatal conductance measurements were obtained by porometry using one portable SC-1 Steady State Leaf Porometer (Decagon Devices, Pullman, WA, United States). Stomatal conductance was measured on the abaxial surface of each leaf once per day between 08:30 h and 14:00 h during the growing season under microclimate conditions prevailing at the time of measurement. On average, we made 60 measurements per day, per site, with an average of five and a half minutes between each measurement. Stomatal conductance was measured on each leaf on three to four consecutive days to purposefully capture variance in *g*_s_ following the variance protocol (McElwain et al., 2016). One leaf from each of three to four individuals per species was measured, thus, each species per site was represented by three to four leaves. The definitions of eight types of stomatal conductance referred to in this manuscript are set out in Table 2.

**Table 2:** List of definitions of eight stomatal conductance (*g*_s_) parameters (all in mmol m^-2^ s^-1^) referred to in this study.

Parameter	Definition
*g*_s_	One *in situ* stomatal conductance to water vapor measurement on the abaxial (lower) surface of one leaf once per day between 08:30 h and 14:00 h under prevailing environmental conditions during the growing season.
*g*_smax_	The highest measured value of *in situ g*_s_ from one species at one site (from an average of 12 measurements, over three to four consecutive days).
*g*_smax(abs)_	The maximum *g*_s_ achievable in the field when species-specific plant growth conditions are at optimum.
*g*_smax(anatomy)_	The absolute theoretical maximum *g*_s_ based on stomatal density and pore size: $$\begin{array}{c} g_{\max}=\frac{\frac{dw}{v}\cdot SD\cdot pa_{\max}}{pd+\frac{\pi }{2}\sqrt{\frac{pa_{\max}}{\pi }}} \end{array}$$ however, beyond this reference description, *g*_smax(anatomy)_ will not be considered further in this paper [refer to McElwain et al. (2016) for a full review of the *g*_smax(anatomy)_ relationship to *g*_s_ and formula explanation]
*g*_smax(day)_	The highest value of all *g*_s_ measurements for a given day between 08:30 h and 14:00 h, for any leaf (regardless of species, selected from an average of 60 measurements).
*g*_s(IRGA)_	Stomatal conductance measured by infra-red gas analysis (IRGA) in this study.
*g*_smax(lit)_	The maximum stomatal conductance to water vapor measurements from published literature (Maire et al., 2015).
*g*_smax(CT)_	The mean or median of maximum stomatal conductance [of either *g*_smax_ or *g*_smax(lit)_] from a representative bioclimatic zone-specific random sample of stomatal conductance measurements that are independent and identically distributed; therefore, *g*_smax(CT)_ represents the sample mean or median of C_3_ woody angiosperm populations across bio-climatic zones and is a measure of the central tendency (the median is used when a distribution is skewed) of either *g*_smax_ or *g*_smax(lit)_.

In the field, stomatal conductance rarely operates at absolute maximum (Schulze et al., 1994; Körner, 1995). This is the absolute maximum *g*_s_ achievable in the field when species-specific plant growth conditions are at optimum [*g*_smax(abs)_] (see Table 2). To estimate *g*_smax(abs)_, we measured *g*_s_ on the same leaf every day for 3–4 days and selected the highest value to estimate *g*_smax(abs)_, which is referred to hereafter as *g*_smax_. This method ensured that we captured as much natural variance as possible within the constraints of the study, however, we acknowledge that it may still not have captured *g*_smax_. Stomatal conductance was measured on the interveinal areolae at mid-lamina of the abaxial surface of healthy, fully expanded, sun-exposed leaves on mature plants of both tall and short stature. No saplings were included in the study. For tall trees we measured the outermost reachable leaves. In the case of compound leaves, the terminal leaflet was measured; for larger leaves the sensor was clamped as far onto the leaf as possible, taking care to avoid damage to the leaf margin. In the subtropical desert, where mid-day depression of stomatal conductance is common, all measurements were taken before mid-day.

Environmental data [air temperature and relative humidity (RH)] were recorded at each leaf, using a thermo-hygrometer (HD2101.2, Delta-Ohm, Padua, Italy), before each *g*_s_ measurement. From these, vapor pressure deficit (VPD) was calculated according to the August-Roche-Magnus formula (Murray, 1967). Photosynthetic photon flux density (PPFD) (expressed as PAR – photosynthetically active radiation – in μmol (photons) m^-2^ s^-1^) was measured using a ceptometer (AccuPAR LP-80, Decagon, Pullman, WA, United States), which was calibrated each morning. Each measurement location was geo-referenced (eTrex^®^ 10, Garmin, Hampshire, United Kingdom) to enable subsequent sourcing of external ancillary data such as local climate.

### Comparison of Porometry-Measured *g*_s_ Data With Published Data Using Infra-Red Gas Analysis (IRGA)

When using a porometer it is important to be aware of a number of factors which could potentially result in elevated stomatal conductance readings. The Decagon SC-1 steady state porometer measures stomatal conductance by putting the conductance of the leaf in series with two known vapor concentration points in the diffusion path inside the porometer clamp. By using the known humidity at three locations in the leaf and at the two humidity sensors in the porometer head, the porometer calculates the resistance between the inside and outside of the leaf. Conditions within the sensor head are steady-state (i.e., non-ventilated), which may affect boundary layer resistance. The sensor head is also fully dark. To compensate for the possible effect of the sensor head environment on stomatal conductance, the porometer takes readings within 30 s. The temperature of the first sensor is assumed to be the same as the temperature of the leaf, therefore it is possible that the non-equilibrium of the sensor head with leaf temperature may affect stomatal conductance readings.

In contrast, infra-red gas analyzers (IRGA) are open systems that control environmental variables such as light, temperature and CO_2_ within the cuvette. Because these two methods use fundamentally different mechanisms to measure stomatal conductance they regularly produce different stomatal conductance values. Therefore, in order to make meaningful comparison and assessment of the STraits stomatal conductance data against published datasets of IRGA-measured stomatal conductance, we also took measurements using an IRGA from a subset of the same porometry-measured STraits species during the same fieldwork campaign to enable a porometry-IRGA data comparison study.

### Stomatal Conductance [*g*_s(IRGA)_] and Microclimate Measurements – Infra-Red Gas Analyzer

A total of 48 species (∼22% of the total 218 species measured by porometry) were measured using a CIRAS-2 gas analyzer (PP-Systems, Amesbury, MA, United States) attached to a PLC6 (U) cuvette fitted with a 1.7 cm^2^ measurement window and a red/white light LED unit. The IRGA study included species from sites in the temperate deciduous forest, boreal forest and tropical seasonal forest (moist). Stomatal conductance was measured on an average of four individuals per species between 09:00 h and 13:00 h. To do this, a 1- to 2-m long sun-exposed branch was excised from each individual following a standard protocol (Dang et al., 1997; Koch et al., 2004; Berveiller et al., 2007; Domingues et al., 2010; Rowland et al., 2015). For species located within the general vicinity of the IRGA harvested branches were immediately recut under water at least 10 cm from the excision point. In cases where individuals were located some distance from the IRGA location, much longer branches were harvested. At the IRGA location these were then cut at approximately 50 cm from the excision point to remove excess material and immediately recut under water at least 10 cm from the second cut. This procedure was followed to safeguard against the formation of embolisms in vessels close to the measured leaf and carried out within 5 min of the initial harvesting (Dang et al., 1997; Koch et al., 2004; Berveiller et al., 2007; Domingues et al., 2010; Rowland et al., 2015). A fully expanded leaf from each branch was enclosed in the cuvette of the gas analyzer and stomatal conductance at ambient CO_2_ concentration (400 ppm) was recorded upon stabilization of its value, which typically took less than 15 min (Betts et al., 2016; Purcell et al., 2018). Air flow, light intensity and incoming mole fraction of water during the measurements were maintained at 200 cm^3^ min^-1^, 1,000 μmol m^-2^ s^-1^ and 80–90% of ambient, respectively. All stomatal conductance measurements were taken under a calculated site-specific mean leaf temperature. This was obtained at 09:00 h on the first measurement day at each site, by running the gas analyzer at the set points stated above (i.e., 1,000 μmol m^-2^ s^-1^ of light, 80–90% of ambient water vapor, 400 μmol mol^-1^ CO_2_) without setting any type of temperature control. The temperature of one leaf from each of 10 randomly selected species (i.e., 10 leaves) growing at the site was then recorded and used for calculating a general average site-specific leaf temperature. Measuring leaves in a chamber changes the thermal environment of the leaves by placing them in a wind stream of relatively high velocity that could bring leaf temperatures close to air temperatures, as they can quickly equilibrate to conditions within the cuvette. For this reason, recording of the leaf temperatures was carried out immediately after clamping the leaves (i.e., within ∼1 s), thus not allowing them to equilibrate in the measurement cuvette, which could result in temperature adjustments due to differences in boundary layer development.

### Scaling Relationship Between Porometry-Measured and IRGA-Measured Stomatal Conductance

**FIGURE 2:**
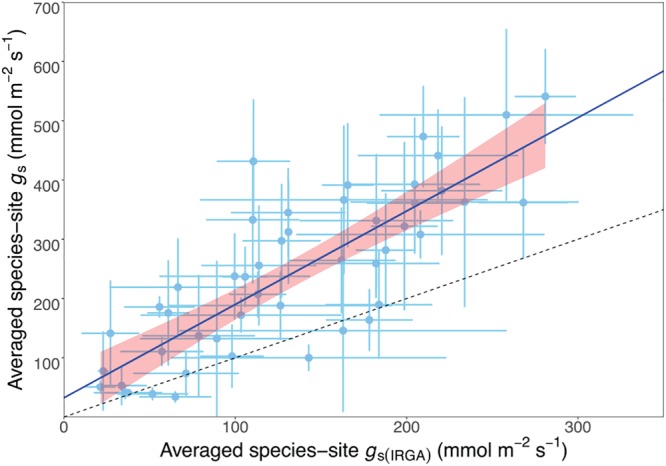
Scatterplot of matching porometer- and infrared gas analysis- (IRGA-) measured species-site (i.e., from the same species at the same site) stomatal conductance (*g*_s_) showing a linear relationship, where the equation average species-site *g*_s_ = 1.42 · average species-site *g*_s(IRGA)_ + 30. *r*^2^ = 0.81, *n* = 48, *P* < 0.01. The dashed line is the 1:1 relationship.

We investigated the relationship between porometry-measured and IRGA-measured stomatal conductance to obtain a correction factor for the STraits *g*_s_ dataset. We plotted the average *g*_s_ from the IRGA-measured data subset of 48 species against the same porometry-measured species and site (hereafter referred to as species-site) from the STraits dataset with a fixed intercept at 30 mmol m^-2^ s^-1^ (Figure 2). This intercept value corresponds to the average stomatal conductance using porometry when measured on a dry filter paper (29.8 + 2.7). The resulting scaling relationship was used to correct all STraits *g*_s_ values greater than 30 mmol m^-2^ s^-1^ using the porometry-IRGA training dataset described:

$$\begin{array}{c} \mathrm{Average}\;species-site\;g_{s}=1.42\;\cdot \mathrm{average}\;species-site\;g_{s(\mathrm{IRGA})}+30\;(Figure\;2), \end{array}$$

and only the corrected *g*_s_ values were used for all analysis. Porometer values equal to and below 30 mmol m^-2^ s^-1^ were discarded (total five *g*_s_ data points).

An additional difference between the porometry and IRGA methods is that porometry measures only one side of a leaf at a time, whereas IRGA systems measure both the lower and upper leaf surfaces simultaneously. Many *Populus* and *Salix* species are amphistomatous, however, some species in this family (Salicaceae) possess no adaxial stomata, and indeed some of the amphistomatous species display a great deal of heterogeneity in the ratio of upper to lower stomatal densities (Binns and Blunden, 1980; Chen et al., 2008) as well as in their behavior. Examination of the leaf specimens in this study confirmed that all specimens in the subset measured by both porometry and IRGA, which were used to generate the scaling relationship, were hypostomatous. Amphistomaty is also a feature of some species from high light environments, such as Mediterranean and subtropical desert plants, some of which are included in this study, [for example, *Baccharis pilularis* DC. and *Simmondsia chinensis* (Link) C.K. Schneid, respectively]; however, since for this study only abaxial (lower surface) stomatal conductance was measured, we were confident in applying the above scaling factor to our STraits *g*_s_ dataset.

### Analysis

#### General Statistical Analysis

Statistical analysis and graphing were carried out using R statistical package Version 3 (R Core Team, 2015). Except for the Generalized Extreme Value analysis (GEV), all analyses were undertaken using *g*_smax_ (430 data points). Data were pooled according to bioclimatic zone, habitat (open-canopy and understory-subcanopy) and habitat within bioclimatic zone. Pooled data within habitats and bioclimatic zones were all approximately normally distributed. We tested for convergence in *g*_smax_ of C3 woody angiosperms between bioclimatic zones using one-way ANOVA and applied a *post hoc* analysis of Tukey’s honest significant difference test to identify differences between pairs of means of bioclimatic zones. For highly skewed data, e.g., PAR and VPD data, a Kruskal–Wallis test was used to test for systematic differences among bioclimatic zones and a Wilcoxon rank-sum test was used to examine pairwise differences between groups. Throughout the paper, the significance threshold was set at 0.05. All errors (±) refer to standard deviation, unless otherwise stated.

To partition the *g*_smax_ variance components, ‘species,’ ‘site,’ and ‘bioclimatic zone,’ in each habitat group, we fitted a model for decomposition of variances consisting of species and site nested in bioclimatic zone using a maximum likelihood method implemented in ‘lme4’ package, ‘lmer’ function (Bates et al., 2015) in ‘R’. The *g*_smax_ values were log_10_-transformed and assigned y_i_ as the log base-10 value of *g*_smax_ observation i = 1,..,n. The following model equation is then

$$\begin{array}{c} y_{i}=\beta_{0}+b_{bioclimatic\;zone_{i},\;site_{i}}+c_{species_{i}}+{𝜖}_{i} \end{array}$$

where 𝜖_i_ ∼ N(0, σ^2^) is a residual error variance, b_bioclimaticzone_i_, site_i__ ∼ N(0, $\sigma_{b}^{2}$) is a site within bioclimatic zone random effect, and c_species_i__ ∼ N(0, $\sigma_{c}^{2}$) is a species random effect; β_0_ is the intercept, *b* and *c* are regression coefficients, 𝜖 is the residual and σ^2^ is the population variance. The syntax in ‘lmer’ function is as follows: log_10_ *g*_smax_ ∼(1| Bioclimatic zone/Site) + (1| Species). The percentage variation explained by random effects were extracted directly from the model output.

#### The Daily Maximum Stomatal Conductance [*g*_smax(day)_] – Generalized Extreme Value Statistics

Generalized extreme value (GEV) theory has been extensively used as an appropriate statistical method in the applied sciences (Coles, 2001). This is likely the first time it has been used in the analysis of stomatal conductance data. The objective of an extreme value analysis is to quantify the stochastic behavior of a process of extreme values, either at very large or small values, i.e., estimation of the probability of events that are more extreme than those already observed. For example, if a large number of independent random values of biological traits, that follow a single probability distribution, were generated, and only the maximum values extracted, then the distribution of those maximum values would be considered as having an approximately GEV distribution.

For this study, the GEV statistical method was appropriate to determine the distribution of maximum values of *g*_smax_, say in blocks of days. We defined *g*_smax_ as the highest measured value of *g*_s_ from one species at one site [from an average of 12 measurements, over three to four consecutive days (Table 2)]. For this reason, and because of the inherent variability in *g*_s_, we cannot be certain that *g*_smax_ as described here is a good proxy for *g*_smax(abs)_ in the field. In this regard, we acknowledge that there is always the possibility that exceptional biotic and/or abiotic factors may influence *g*_s_ values. Given that *g*_s_ rarely operates at *g*_smax(abs)_ (Schulze et al., 1994; Körner, 1995), the occurrence of a plant conducting at its absolute maximum in natural field conditions may be viewed as an extreme event under the framework of GEV theory. We used GEV theory to validate the robustness of our approach to determining *g*_smax_ by fitting *g*_smax(day)_ values to the GEV distribution and calculating a modeled range of extreme *g*_s_ values for the total dataset and for the open-canopy and understory-subcanopy groups. Greater than 50% of the *g*_smax_ values were higher than the lower limit of the 95% confidence interval (CI) of the GEV fitted *g*_smax(day)_ data, therefore, we can be confident that the *g*_smax_ values presented here are substantially representative of *g*_smax(abs)_. For GEV analysis, we used the highest value of *g*_s_ obtained in a given day [*g*_smax(day)_] from a pool of, on average, 60 measurements per day from across all leaves and species, amounting to a total *g*_smax(day)_ sample of 74 data points (i.e., 74 measurement days) [see *g*_smax(day)_ definition in Table 2].

A common way of dealing with extreme values in GEV analysis is to use the block maxima (BM) method, that is, to retain only the maximum observed value in a specified period, e.g., the daily maximum, given that there would be many observations in that period. We used the BM method to model the maximum stomatal conductance of any leaf, regardless of species, over a specified period. It is reasonable to assume that the *g_s_* of different woody angiosperm species across all sites will follow a common probability distribution, since stomatal conductance as a physiological process is governed by the same principles in all vegetation. Our model focused on the statistical behavior of

$$\begin{array}{c} g_{\mathrm{smax}(\mathrm{day})m}=\max\{g_{s1},g_{s2},\ldots .g_{sn}\}, \end{array}$$

where *m* = 1st, 2nd, 3rd,…74th day and *g*_s1_, *g*_s2_,….*g*_s_*_n_* is a sequence of independent random stomatal conductance (*g*_s_) measurements for any leaf, regardless of species, having a common distribution, and *n*-observations were measured at regular intervals of on average five and a half minutes each between 08.30 and 14.00 h per site. Therefore, *g*_smax(day)_*_m_* above represents the highest *g*_smax_ on *m*th day for any leaf [see definitions of *g*_smax_ and *g*_smax(day)_ in Table 2]. For the total dataset, the number of *n*-observations was on average 60 per day per site, while for each of the habitats the number of *n*-observations was on average 30 per day per site.

We fitted the *g*_smax(day)_ values into a GEV distribution using a maximum likelihood estimation (Coles, 2001) implemented in R package ‘ismev’ (Heffernan and Stephenson, 2001) for the total dataset and for each of the open-canopy and understory-subcanopy habitat groups. Subsequently, the 95% CI was calculated for each of the three groups of data. The lower and upper limits of the 95% CI were treated as estimates of the greatest possible range of *g*_s_ for a given day.

#### Analysis of Maximum Stomatal Conductance From Published Literature [*g*_smax(lit)_]

For comparison of our data with the published literature, we extracted only the C_3_ woody angiosperm species (deciduous and evergreen trees and shrubs) data from the data compilation of Maire et al. (2015). Stomatal conductance in Maire et al. (2015) was measured under ambient field conditions using IRGA during daytime hours during the growing season. All stomatal conductance measurements were taken under high light conditions (PAR between 580 and 1,540 μmol m^-2^ s^-1^) and, for most of the values presented, had already been averaged by species and site. For comparison purposes, the Maire et al. (2015) dataset is referred to as *g*_smax(lit)_ (Table 2). We calculated the statistics (moments) and generated kernel density plots for *g*_smax(lit)_ from Maire et al. (2015) and the *g*_smax_ from STraits. The mean and median (i.e., central tendency) values of both datasets are termed *g*_smax(CT)_ (see Table 2 for definition). We also compared the *g*_smax(lit)_ of the same species in sites or bioclimatic zones common to both the Maire et al. (2015) and this study.

## Results

The 2013–2015 field campaign resulted in the primary ‘STraits’ dataset of 4273 individual *g*_s_ measurements of 218 C3 woody angiosperm species, representing 60 families sampled across seven bioclimatic zones. Individual *g*_s_ data were corrected using the porometry-IRGA scaling relationship calibration equation. 430 estimated *g*_smax_ values were generated, of which 217 were from the open-canopy habitat and 213 from the understory-subcanopy habitat (see Supplementary Material). This also amounted to a total of 74 daily maximum *g*_s_ values, *g*_smax(day)_.

**FIGURE 3:**
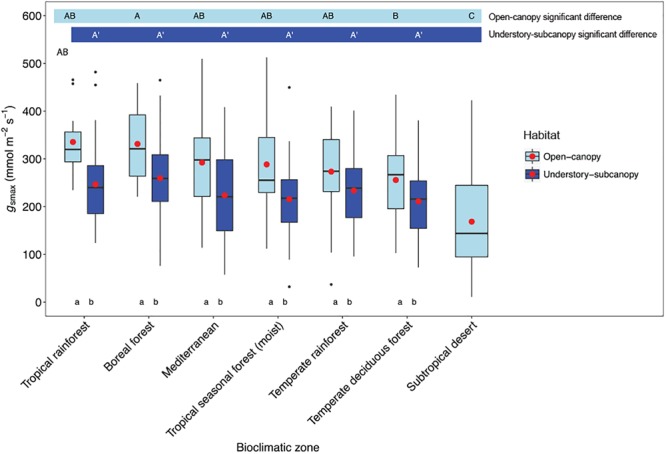
Boxplots comparing maximum stomatal conductance (*g*_smax_) in the open-canopy and understory-subcanopy habitats across bioclimatic zones. Boxplots are arranged from highest to lowest average *g*_smax_ according to the open-canopy habitat. Capital letters above boxplots indicate pairwise comparison across bioclimatic zones using Tukey’s honest significant difference (*P* < 0.05) for the open-canopy (first row, without apostrophe) and the understory-subcanopy (second row, with apostrophe), (the same letter means the variables are not significantly different while a different letter means they are significantly different). ANOVA was used to analyze differences across bioclimatic zones. Boxes represent the interquartile range (IQR), horizontal line within boxes represents the median, the red dot represents the mean and whiskers extend to 1.5 times the IQR; black dots are outliers.

Considering understory-subcanopy data only, there was clear convergence in *g*_smax_ of C3 woody angiosperms across bioclimatic zones, that is, there was no significant difference in mean *g*_smax_ [ANOVA: *F*(5,207) = 1.91, *P* = 0.09] (Supplementary Table S1). Mean *g*_smax_ in the understory-subcanopy habitat was significantly lower than that of open-canopy habitat in six bioclimatic zones (the subtropical desert included only open-canopy habitat taxa in this study) (Figure 3 and Table 3). The interquartile range of understory-subcanopy *g*_smax_ varied between 150 and 309 mmol m^-2^ s^-1^ (Figure 3 and Table 3).

**Table 3:** Summary statistics of maximum stomatal conductance (*g*_smax_, mmol m^-2^ s^-1^) for the understory-subcanopy habitat in six bioclimatic zones.

Bioclimatic zone	*n*	Spp.	Mean	*SD*	Median	Max	Min	1st quartile	3rd quartile	*L*(*K*–*S*)	*P*-value of two-sample *t*-test between habitat groups
Boreal forest	41	21	260^A^	90	259	465	76	211	309	0.09^∗^	<0.001
Mediterranean	24	20	224^A^	100	221	409	58	150	298	0.07^∗^	0.007
Temperate deciduous forest	53	37	211^A^	75	216	381	73	154	254	0.10^∗^	0.006
Tropical rainforest	40	30	247^A^	85	240	482	124	186	286	0.10^∗^	0.001
Temperate rainforest	37	22	234^A^	81	239	401	96	177	280	0.06^∗^	0.030
Tropical seasonal forest (moist)	18	18	216^A^	93	218	450	32	167	256	0.13^∗^	0.002

*n, number of species-site observations; SD, standard deviation; L(K–S), Lilliefors (Kolmogorov–Smirnov) test for departure from normality. ^*A*^Values sharing the same letter are not significantly different by Tukey’s honest significant difference, see Supplementary Table S1 for P values of pairwise comparison. ^∗^P < 0.05, data is significantly different from normal distribution.*

There was no convergence in *g*_smax_ of C3 woody angiosperms across bioclimatic zones in the open-canopy habitat [ANOVA: *F*(6,250) = 12.5, *P* < 0.001] (Supplementary Table S2). We observed significant difference in mean open-canopy *g*_smax_ between the boreal forest and the temperate deciduous forest (Figure 3, Table 4, and Supplementary Table S2). The open-canopy habitat of the subtropical desert demonstrated the lowest overall mean *g*_smax_ at 169 mmol m^-2^ s^-1^, significantly different from the mean *g*_smax_ in all other bioclimatic zones (Figure 3, Table 4, and Supplementary Table S2). The interquartile range of the open-canopy *g*_smax_ varied between 95 and 392 mmol m^-2^ s^-1^. In the understory-subcanopy habitat the highest *g*_smax_ values across bioclimatic zones ranged from 401 to 482 mmol m^-2^ s^-1^ (Figure 3 and Table 3). In the open-canopy habitat highest *g*_smax_ values ranged between 410 and 513 mmol m^-2^ s^-1^ (Figure 3 and Table 4).

**Table 4:** Summary statistics of maximum stomatal conductance (*g*_smax_, mmol m^-2^ s^-1^) for the open-canopy habitat in all seven bioclimatic zones.

Bioclimatic zone	*n*	Spp.	Mean	*SD*	Median	Maximum	Minimum	1st quartile	3rd quartile	*L*(K–S)
Boreal forest	24	14	331^A^	72	321	459	221	264	392	0.12^∗^
Mediterranean	47	33	292^AB^	90	298	510	114	221	344	0.07^∗^
Subtropical desert	38	18	169^C^	99	144	423	11	95	245	0.12^∗^
Temperate deciduous forest	45	30	256^B^	81	267	434	103	196	307	0.06^∗^
Tropical rainforest	12	11	335^AB^	69	320	466	235	294	357	0.17^∗^
Temperate rainforest	45	26	273^AB^	81	274	410	37	232	341	0.06^∗^
Tropical seasonal forest (moist)	6	6	289^AB^	138	255	513	112	229	345	0.13^∗^

*n, number of species-site observations; SD, standard deviation; L(K–S), Lilliefors (Kolmogorov–Smirnov) test for departure from normality. ^*A,B,C*^The same letter means the variables are not significantly different while a different letter means they are significantly different by Tukey’s honest significant difference, see Supplementary Table S2 for P-values of pairwise comparison. ^∗^P < 0.05, data is significantly different from normal distribution.*

**FIGURE 4:**
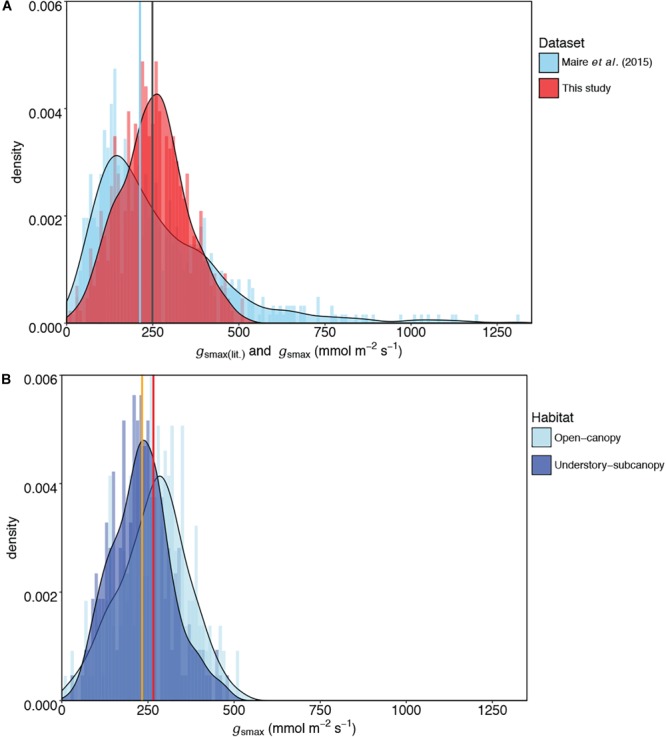
Comparisons of kernel density plots of maximum stomatal conductance (*g*_smax_) for **(A)** this study and published literature [*g*_smax(lit)_] (Maire et al., 2015), and **(B)** the open-canopy habitat and understory-subcanopy habitat. In **(A)** the central tendencies of each dataset are indicated by vertical lines in blue (median) for Maire et al. (2015) and gray (mean) for this study; in **(B)** the vertical orange line indicates the mean *g*_smax_ of the understory-subcanopy habitat and the red line the mean *g*_smax_ of the open-canopy. For visual readability, the *x*-axis in **(A)** was limited to 1,350 mmol m^-2^ s^-1^ instead of the maximum value of 2,272 mmol m^-2^ s^-1^ in Maire et al. (2015) dataset (see Table 5).

**FIGURE 5:**
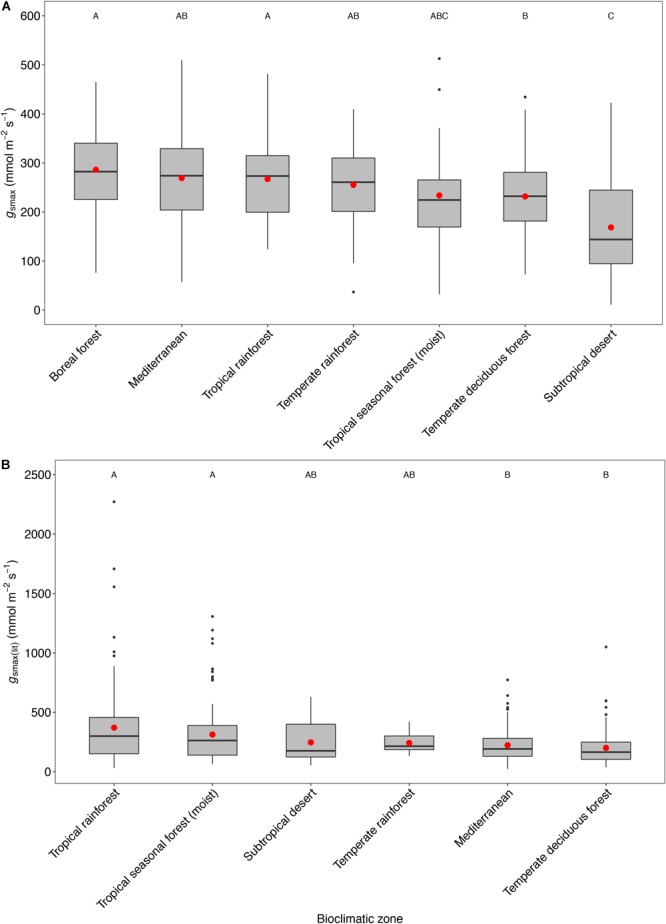
Boxplots comparing maximum stomatal conductance across bioclimatic zones for this study **(A)** in combined open-canopy and understory-subcanopy *g*_smax_ data and for published literature **(B)** from Maire et al. (2015) for *g*_smax(lit)_ data. Boxplots are arranged from the highest to the lowest average value. Capital letters above boxplots indicate pairwise comparison across bioclimatic zones using Tukey’s honest significant difference (*P* < 0.05), (the same letter means the variables are not significantly different while a different letter means they are significantly different). ANOVA was used to analyze differences across bioclimatic zones. Boxes represent the interquartile range (IQR), horizontal line within boxes represents the median, the red dot represents the mean and whiskers extend to 1.5 times the IQR; black dots are outliers.

The mean *g*_smax_ of the total dataset was 249 mmol m^-2^ s^-1^ (±95) (Table 5). There was a significant difference in mean *g*_smax_ between the open-canopy (266 mmol m^-2^ s^-1^ ± 100) and understory-subcanopy habitat (233 mmol m^-2^ s^-1^ ± 86) (*P* < 0.001) (Table 6). The density distributions of *g*_smax_ for the total dataset (Figure 4A and Table 5) and for both open-canopy and understory-subcanopy groups (Figure 4B and Table 6) all displayed a normal distribution. Therefore, the central tendency for the total dataset was a mean *g*_smax_ of 249 mmol m^-2^ s^-1^ (Table 5). By comparison, the compiled data from Maire et al. (2015) displayed a skewed distribution following a log-normal distribution (Figure 4A). The central tendency for this dataset with skewed distribution was the median *g*_smax_, 211 mmol m^-2^ s^-1^ (Table 5). There was no significant difference between the mean value observed in Maire et al. (2015) and that in STraits (*P* = 0.077), with mean *g*_smax(lit)_ and *g*_smax_ values of 268 and 249 mmol m^-2^ s^-1^, respectively (Figure 4A and Table 5). The Maire et al. (2015) dataset includes *g*_smax(lit)_ data from two separate studies of Jasper Ridge Biological Preserve (Field, 1983; Ackerly, 2004), which is the only site common to both Maire and our study. Comparison of 12 species from Jasper Ridge common to both Maire et al. (2015) and STraits studies shows that the *g*_smax_ captured in STraits was higher than that presented in Maire et al. (2015) by ∼2% (Ackerly, 2004) and ∼16% (Field, 1983) (Supplementary Table S3). STraits *g*_smax_ values were on average 22% higher than in Maire et al. (2015) for 26 species common to both studies and from the same bioclimatic zones (Supplementary Table S4). In a combined open-canopy and understory-subcanopy habitat analysis, there was no evidence of overall convergence in *g*_smax_ of C3 woody angiosperms among bioclimatic zones [ANOVA: *F*(6,423) = 8.66, *P* < 0.001] (Figure 5A and Supplementary Tables S5, S6). This is in agreement with Maire et al. (2015) [ANOVA: *F*(5,561) = 9.89, *P* < 0.001] (Figure 5B and Supplementary Tables S7, S8). A comparison of maximum stomatal conductance data for C3 woody angiosperms from published datasets and this study is presented in Supplementary Table S9 and shows no obvious trend in lowest to the highest average biome/bioclimatic zone *g*_smax(lit)_ or *g*_smax_ amongst datasets.

**Table 5:** Summary statistics of maximum stomatal conductance (*g*_smax_, mmol m^-2^ s^-1^) of STraits and the C_3_ woody angiosperm *g*_smax_ data subset from Maire et al. (2015) [*g*_smax(lit)_].

Dataset	*n*	Spp.	Mean	*SD*	Median	Minimum– maximum	1st quartile	3rd quartile	Skewness	Kurtosis	*L*(*K–S*)	Distribution^1^
STraits	430	217	249^A^	95	252	11–513	183	309	0.01	2.75	0.03	Normal
Maire et al., 2015	567	473	268^A^	221	211	24–2272	133	348	3.26	21.32	0.14^∗^	Log-normal

*n, number of species-site observations; SD, standard deviation; L(K-S), Lilliefors (Kolmogorov-Smirnov) test for departure from normality. ^*1*^Distribution model with lowest AIC values selected after each dataset was fitted with both normal and log-normal distribution models. ^*A*^Values sharing the same letter are not significantly different by two-sample Student’s t-test, P = 0.077. ^∗^P < 0.05, data is significantly different from normal distribution.*

**Table 6:** Summary statistics of maximum stomatal conductance (*g*_smax_, mmol m^-2^ s^-1^) for the understory-subcanopy and open-canopy habitats from the STraits dataset.

Habitat	*n*	Spp.	Mean	*SD*	Median	Minimum–maximum	1st Quartile	3rd Quartile	Skewness	Kurtosis	*L(K–S)*	Distribution^1^
Open-canopy	217	123	266^A^	100	274	11–513	200	329	0.15	2.76	0.05^∗^	Normal
Understory-subcanopy	213	139	233^B^	86	232	32–482	175	282	0.31	2.99	0.04^∗^	Normal

*n, number of species-site observations; SD, standard deviation; L(K-S), Lilliefors (Kolmogorov-Smirnov) test for departure from normality. ^*1*^Distribution model with lowest AIC values selected after each dataset was fitted with both normal and log-normal distribution models. ^*A,B*^Values with different letter are significantly different by two-sample Student’s t-test, P < 0.001. ^∗^P < 0.05, data is significantly different from normal distribution.*

**FIGURE 6:**
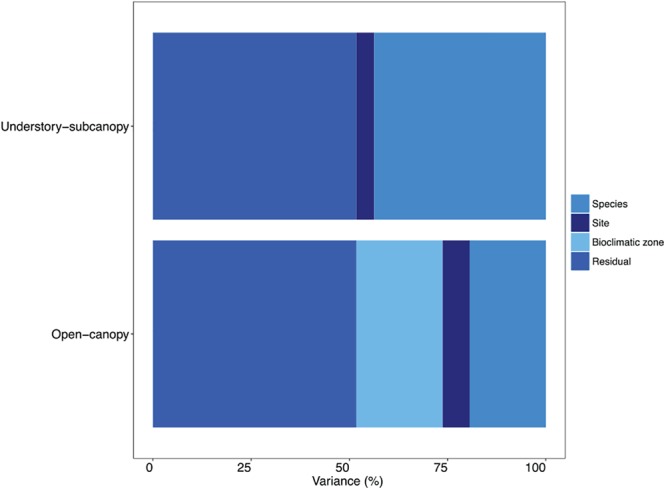
Bar graph showing in percentages the proportional contribution of species, site, bioclimatic zone and residual to variance in maximum stomatal conductance (*g*_smax_) in the understory-subcanopy and open-canopy habitats (see Supplementary Table S10 for percentage breakdown).

A variance component analysis of the STraits *g*_smax_ dataset summarizes in percentages the contribution to overall variance in *g*_smax_ by species, site and bioclimatic zone for the two habitat groups (Figure 6 and Supplementary Table S10). In the understory-subcanopy group the contribution by bioclimatic zone was negligible, which is in contrast to the open-canopy where it contributes 22% to overall variance. The variance contribution by species in the understory-subcanopy is more than double that in the open-canopy (44 and 19%, respectively).

**FIGURE 7:**
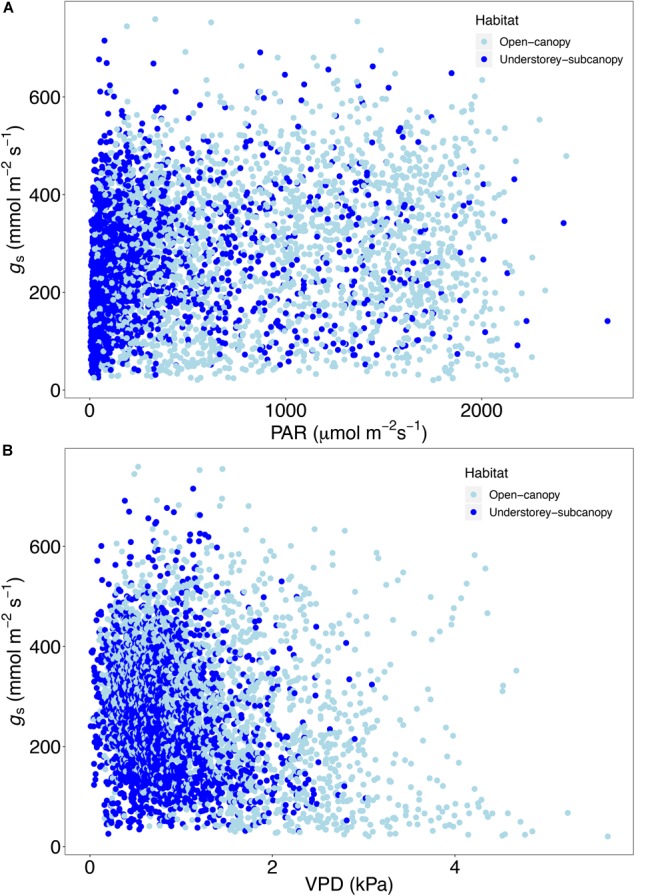
Scatter plot of stomatal conductance (*g*_s_) (*n* = 4273) versus **(A)** photosynthetically active radiation (PAR) and **(B)** vapor pressure deficit (VPD) in the open-canopy and understory-subcanopy habitats.

**FIGURE 8:**
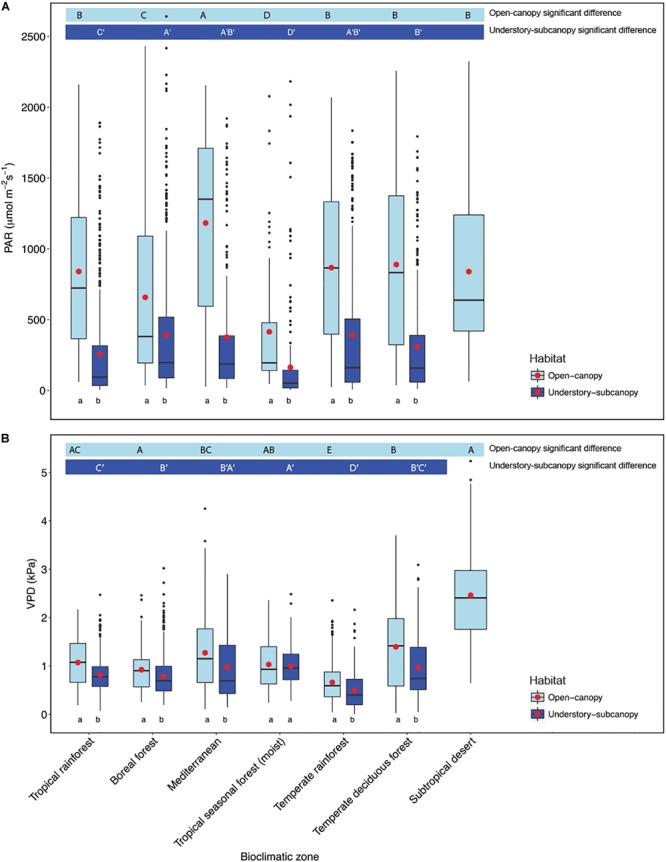
Boxplots comparing **(A)** photosynthetically active radiation (PAR) and **(B)** vapor pressure deficit (VPD) of the open-canopy and understory-subcanopy habitats in each bioclimatic zone. Boxplots are arranged following Figure 3, i.e., from the highest to the lowest average *g*_smax_ according to open-canopy habitat. Capital letters above boxplots designate pairwise comparison across bioclimatic zones using Wilcoxon rank-sum with Bonferroni correction (*P* < 0.05) for open-canopy (without apostrophe, first row) and understory-subcanopy (with apostrophe, second row), (the same letter means the variables are not significantly different while a different letter means they are significantly different). Kruskal–Wallis test was used to analyze differences across bioclimatic zones. Lower-case letters below boxplots indicate comparison of habitats in the same bioclimatic zone by two-sample Wilcoxon rank-sum. Boxes represent the interquartile range (IQR), horizontal line within boxes represents the median, the red dot represents the mean and whiskers extend to 1.5 times the IQR; black dots are outliers.

Scatterplots of *g*_s_ versus both PAR and VPD showed considerable overlap of open-canopy and understory-subcanopy values (Figure 7), with *g*_s_ in both habitats covarying greatly with PAR and VPD. However, there was significant difference in both PAR and VPD values between bioclimatic zones in both habitats, with the understory-subcanopy PAR and VPD values displaying generally lower values than in the open-canopy habitat across most bioclimatic zones (Figure 8 and Supplementary Tables S11–S18 inclusive). Only VPD values in the tropical seasonal forest (moist) showed no significant difference between habitats (Figure 8B) (*P* = 0.90). The understory-subcanopy habitat demonstrated less variability in both PAR and VPD than the open-canopy between bioclimatic zones (Supplementary Tables S11–S18 inclusive). The subtropical desert displayed generally higher VPD values than other bioclimatic zones, with mean VPD there nearly double that observed in most other bioclimatic zones (Figure 8B and Supplementary Tables S17, S18).

All *g*_smax(day)_ values (i.e., for the entire dataset and the open-canopy and understory-subcanopy habitat datasets) demonstrated good fit to the GEV distribution (see Supplementary Figure S1 for diagnostics and Supplementary Table S19 for 95% CI parameters). All datasets fell into a type III extreme value distribution, i.e., Weibull distribution, based on the negative shape of the parameter values. Based on the GEV distribution analysis of the entire dataset, the lower and upper limits of the 95% CI *g*_smax(day)_ were 233 and 484 mmol m^-2^ s^-1^, respectively. The open-canopy habitat lower and upper limits of the 95% CI *g*_smax(day)_ were 226 and 481 mmol m^-2^ s^-1^, respectively, and the understory-subcanopy lower and upper limits of the 95% CI *g*_smax(day)_ were 181 and 456 mmol m^-2^ s^-1^, respectively (Supplementary Table S19). The total dataset and the open-canopy and understory-subcanopy habitat groups each demonstrated that >50% of their *g*_smax_ were above the lower limit of the 95% CI, at 85, 92, and 89%, respectively, (Supplementary Table S19), indicating that the approach we have taken to determine *g*_smax_ as an approximation of maximum stomatal conductance was robust.

## Discussion

We observed strong convergence in mean *g*_smax_ of C_3_ woody angiosperms in the understory-subcanopy habitat across six of the bioclimatic zones, despite their different plant species and highly distinctive climates. Furthermore, variance analysis revealed that in the understory-subcanopy habitat ‘bioclimatic zone’ as a variance factor had little effect on *g*_smax_ variance, whereas ‘species’ accounted for almost half of overall variance (Figure 6). This suggests that, at the leaf level, plants in the more stable environment of understory-subcanopy habitat are buffered against macroclimate effects (that is, the overall climate of the bioclimatic zone), such as irradiance, temperature and precipitation. A study by De Frenne et al. (2013) on the moderating effect of microclimate on plant responses under macroclimate warming reported a buffering of understory vegetation from macroclimatic effects due to canopy closure and an induced climatic lag in this habitat (De Frenne et al., 2013). This suggestion is also supported by Kamakura et al. (2018) who reported uniform stomatal behavior in leaves in the subcanopy or understory of a Malaysian lowland dipterocarp forest, and where stomatal patchiness in homobaric and heterobaric leaves in the understory environment was similar. They partially attributed the uniformity in stomatal conductance to the less pronounced environmental conditions of irradiance and VPD in the understory than those experienced in the canopy (Kamakura et al., 2018). Our observed trend in the understory-subcanopy *g*_smax_ may reflect a fundamental difference in sensitivity to the effects of macroclimate between the open-canopy and the understory-subcanopy habitats, as classified in this study.

The STraits dataset also demonstrates that there is a central tendency (mean and median) of C_3_ woody angiosperms to operate toward a *g*_smax(CT)_ of ∼250 mmol m^-2^ s^-1^ (Figure 4A and Table 5). The generality in mean and range of *g*_smax_ across bioclimatic zones is interesting given the high number of species the dataset contains and the fact that these *g*_smax_ values were determined by potentially high inherent variability in stomatal density and size within individuals and species. Work is ongoing to determine stomatal morphological traits on the same measured leaves. The central tendency of ∼250 mmol m^-2^ s^-1^ agrees well (i.e., no significant difference) with the mean *g*_smax(lit)_ of Maire et al. (2015) (Table 5) and this is a compelling result given the mostly different C_3_ woody angiosperm species of that meta-analysis. This suggests a strong collective tendency of the C_3_ woody angiosperm species in this study to operate around the mean across six bioclimatic zones (Figure 4A). The observed tendency toward converging *g*_smax_ in C3 woody angiosperm taxa, despite wide geographic and climatic variation and spatial and temporal heterogeneity in stomatal behavior between species, may indicate an emergent property. It suggests a collective response of C3 woody angiosperm species across diverse bioclimatic zones to optimize stomatal conductance in response to constantly shifting environmental and climatic conditions (Mott and Buckley, 2000; Lawson and Blatt, 2014). Our aim in this study was to gather as much stomatal conductance data from as wide a range of C_3_ woody angiosperm species as possible to be representative of a bioclimatic zone, across multiple bioclimatic zones and within the obvious time and resource constraints of such a far-reaching experiment. For these reasons, it was not within the scope of our study to gather abundance data.

Compared to the C_3_ woody angiosperm data subset from Maire et al. (2015), where the data follow a log-normal distribution, the STraits dataset follows a normal distribution pattern (Figure 4A). While most plant traits are known to be log-normally distributed, there are some which are not actually normal on the log scale (Kattge et al., 2011). It has also been suggested that the right-skewness in a given trait distribution is due to the influence of a lower bound to near zero of a particular trait (Kattge et al., 2011); this was not evident from our study. The distribution pattern of the Maire et al. (2015) data subset may be due either to the inclusion of some species with extreme *g*_smax(lit)_ values that reflect the majority difference in species composition, or, to the random effect of different studies using different sampling protocols within their meta-analysis. The attributing of a ‘different studies effect’ to the distribution skewness of Maire et al. (2015) is difficult to test, however, because there are few species or sites in common between the studies. While the STraits dataset also includes species known for high *g*_smax_ (e.g., *Salix* spp.), this does not appear to affect the distribution pattern of the data. Evidence from our comparisons of those species, sites or bioclimatic zones which are common to both Maire et al. (2015) and STraits, together with GEV analysis, confirm that our study has not underestimated *g*_smax_ (Tables 5, 6). We suggest that the use of a standardized protocol by one research team in a single study reduces the random effect of multiple protocols across many different studies. Compilation data can span several decades up to the present time, over which time there may be a rise in atmospheric CO_2_ concentration of up to ∼50 ppm (Supplementary Table S9). It has been shown that even this level of increase can effect biome-level stomatal conductance (Purcell et al., 2018).

We also compared the STraits and Maire et al. (2015) datasets at the level of bioclimatic zone (Figure 5). To do this, it was necessary to lump together the separate open-canopy and understory-subcanopy data from our dataset, since habitat-level data were not available in Maire et al. (2015). While results showed no evidence of convergence in *g*_smax_ of C3 woody angiosperms at bioclimatic zone level between the two datasets, and no similarity in trends of the highest and lowest average *g*_smax_ across bioclimatic zones, we stop short of drawing any conclusions from this comparison for the three reasons outlined in our introduction.

Notwithstanding the observed generality in *g*_smax_ across bioclimatic zones, we have also determined that interspecific difference in *g*_s_ contributes significantly to the variation in *g*_smax_ in both habitats (Figure 6). Community and functional trait-based ecologists widely recognize the importance of interspecific variance (Hulshof and Swenson, 2010; Violle et al., 2012) in field study analyses. Between 60 and 98% of all variation in current plant trait data repositories is accounted for by interspecific variation (Kattge et al., 2011). In a recent study of stomatal conductance in 11 tropical and sub-tropical woody species, “plant identity” (species/plant functional type) was one of the two greatest drivers of *g*_s_ (Tobin and Kulmatiski, 2018).

Light intensity was found to be a major determinant of the observed difference in *g*_smax_ between the open-canopy and understory-subcanopy habitat groups, which was significantly and consistently higher in the open-canopy compared to the understory-subcanopy across six bioclimatic zones (Figure 5A). This agrees with results from a study of microclimate gradients across a New Zealand rainforest edge where summer daily average in-forest PAR was reported to be only ∼0.7% of PAR in open pasture, although in-forest PAR was variable with forest gaps (Davies-Colley et al., 2000). Although VPD is also significantly different in each habitat group in all bioclimatic zones (Figure 5B), it does not appear to have the same influential effect on *g*_s_ as light, as it does not demonstrate the expected inverse relationship with *g*_s_. For example, although we found that VPD in the open-canopy was higher than in the understory-subcanopy, *g*_smax_ was also higher in the open-canopy than in the understory-subcanopy, which is contrary to the expected inverse *g*_s_-VPD relationship. Overall, our observations of PAR and VPD patterns in the open-canopy and understory-subcanopy habitats agree with those reported by Davies-Colley et al. (2000) on the contrasting differences in light and VPD levels between open- and in-forest environments, with less fluctuation of these factors demonstrated in-forest. This said, in this study, plants in the subtropical desert bioclimatic zone, all classified as open-canopy, displayed much lower *g*_smax_ compared to all other bioclimatic zones (Figure 3); however, VPD was found to be twice as high in the subtropical desert as in all other bioclimatic zones (Figure 5B), thus strongly limiting *g*_smax_ of the woody vegetation in this bioclimatic zone. Water availability may also have been a factor in the low *g*_s_ observed in the subtropical desert, however, we believe this is unlikely since we took measurements under optimum conditions during the monsoon season. We did not measure soil moisture at the time of *g*_s_ measurements, however, so we cannot confirm soil moisture levels in this instance.

The abiotic factors tested in this study (PAR and VPD) varied less in the understory-subcanopy than the open-canopy environment. The generally more uniform microenvironment observed in the understory-subcanopy compared to the open-canopy across bioclimatic zones (De Frenne et al., 2013), resulted perhaps in less pronounced differences in *g*_smax_ in the understory-subcanopy habitat across bioclimatic zones. We suggest that this similarity in *g*_smax_ in the understory-subcanopy is due to the shielding of this habitat by the canopy vegetation from the influence of macroclimatic. As a result, the taxa in the understory-subcanopy may be adapted to abiotic factors which fluctuate less, with a lesser effect on *g*_smax_. That said, in the open-canopy habitat, where bioclimatic effect contributed 22% to *g*_smax_ variance, we observed a generality in mean *g*_smax_ in five out of the seven bioclimatic zones investigated.

## Conclusion

The STraits *g*_smax_ data signal a strong convergence in the maximum stomatal conductance of C_3_ woody angiosperms in the understory-subcanopy habitat across large latitudinal gradients. This pattern may be due in part to a buffering against bioclimatic (macroclimatic) effect in this habitat compared to that in the open-canopy habitat. The differential effect of macroclimate on woody vegetation in these two habitats may have implications for the ecophysiological functioning of woody plant communities in different habitats under future macroclimate warming. We expect that woody vegetation in the open-canopy will be more susceptible to future climate change than the understory-subcanopy vegetation and suggest that it will be important for future comparative studies to categorize species based on light availability and/or position in relation to forest canopy. By adopting a standardized protocol in a single study over a relatively short time span, and by categorizing vegetation into habitat groups, this study offers fresh insight into the variability of *g*_smax_ in C_3_ woody angiosperms in natural forest ecosystems, greatly expanding current understanding of maximum stomatal conductance trends across major bioclimatic zones. The STraits dataset will also serve as an important new reference dataset of contemporary *g*_s_ and *g*_smax_ values across wide latitudinal and bioclimatic gradients to advance contemporary (Lawson and Weyers, 1999; Wright et al., 2005; Kattge et al., 2011; Lawson and Blatt, 2014; Kunstler et al., 2016) and paleo (Wilson et al., 2015, 2017; McElwain et al., 2016; Montañez et al., 2016) stomatal conductance research. It will bolster paleoecological and paleoenvironmental studies currently relying on *g*_s_ data from meta-analyses to benchmark *g*_s_ values inferred from fossil plant taxa, both living and extinct (Wilson et al., 2015, 2017; Richey et al., 2018). Indeed, the STtraits dataset also suggests that paleo studies which include sufficient sampling of fossil taxa will likely achieve a robust estimate of paleo *g*_smax_, irrespective of taxa sampled. Such a ‘taxon-free’ approach to estimating paleo-*g*_smax_ opens up the possibility of including extinct taxa, thereby greatly extending the deep time record of *g*_smax_, its evolution and its variation over time. We have also shown for the first time that there is a reliable scaling relationship between the *g*_s_ values obtained by porometry and those by IRGA that can be used to cross-calibrate porometry-measured and IRGA-measured *g*_s_ datasets in future studies. Large trait datasets are observed to be vital links between ecosystem modeling and functional, structural, and adaptive properties of those ecosystems to climate change (Kattge et al., 2011; Garnier and Navas, 2012; Gao et al., 2013; Garnier et al., 2017). Co-ordinating and integrating established but disparate ‘big data’ plant trait datasets is currently underway (Garnier et al., 2017) and thus, the STraits dataset of stomatal conductance is an important addition to a determined effort to model natural vegetation and ecosystem response to environmental change.

## Author Contributions

MM, WS, and JM designed the study and collected and interpreted the STraits data. CY contributed the IRGA data and IRGA-porometry scaling relationship. WS, AP, and MM analyzed the data. MM, WS, and JM (with feedback from CY, SB, RS, TL, RC, IW, and CP) wrote the manuscript. MM and WS contributed equally to the writing of the manuscript.

## Conflict of Interest Statement

The authors declare that the research was conducted in the absence of any commercial or financial relationships that could be construed as a potential conflict of interest.
