# Supplementary material for: Convergence in Maximum Stomatal Conductance of C3 Woody Angiosperms in Natural Ecosystems Across Bioclimatic Zones
Source: Front Plant Sci. 2019 May 7;10:558. doi: 10.3389/fpls.2019.00558 (PMC6514322; doi:10.3389/fpls.2019.00558)
Supplement: Supplementary file 1 [file Table_1.docx]

Supplementary Material

**Convergence in Maximum Stomatal Conductance of C_3_ Woody Angiosperms in Shaded Habitats of Natural Ecosystems Across Six Bioclimatic Zones**

**Michelle Murray^1†*^, Wuu Kuang Soh^1†^, Charilaos Yiotis^2^, Sven Batke^3^, Andrew Parnell^4^, Robert A. Spicer^5^, Tracy Lawson^6^, Rodrigo Caballero^7^, Ian J. Wright^8^_,_ Conor Purcell^1^ and Jennifer C. McElwain^1^**

**Supplementary Figure S1**Diagnostic plots indicating goodness of fit of the Generalized Extreme Value (GEV) distribution to *g*_smax(day)_

**Supplementary Table S1** Post-hoc test by Tukey’s honest significant difference, showing the *P* values of pairwise comparison between mean *g*_smax_ of bioclimatic zones in understory-subcanopy habitat

**Supplementary Table S2** Post-hoc test by Tukey’s honest significant difference, showing the *P* values of pairwise comparison between mean *g*_smax_ of bioclimatic zones in open-canopy habitat

**Supplementary Table S3** Comparison of *g*_smax_ (mmol m^-2^ s^-1^) recorded at Jasper Ridge Biological Preserve for species common to both Maire et al. (2015) and STraits datasets, showing percentage difference in values

**Supplementary Table S4** Comparison of the *g*_smax_/*g*_smax(lit)_ (mmol m^-2^ s^-1^) of common species from the same bioclimatic zones from the STraits dataset and Maire et al. (2015) dataset, showing percentage difference in values

**Supplementary Table S5** Summary statistics of maximum stomatal conductance (*g*_smax,_ mmol m^-2^ s^-1^) for seven bioclimatic zones in this study for combined open-canopy and understory-subcanopy habitat

**Supplementary Table S6** Post-hoc test by Tukey’s honest significant difference, showing the *P* values of pairwise comparison between mean *g*_smax_ of bioclimatic zones in combined understory-subcanopy and open-canopy habitat

**Supplementary Table S7** Summary statistics of maximum stomatal conductance (*g*_smax(lit),_ mmol m^-2^ s^-1^) for six bioclimatic zones in Maire et al. (2015). No data is available for woody angiosperm in the boreal bioclimatic zone.

**Supplementary Table S8** Post-hoc test by Tukey’s honest significant difference, showing the *P* values of pairwise comparison between mean *g*_smax_ of bioclimatic zones in Maire et al. (2015)

**Supplementary Table S9** Comparison of maximum stomatal conductance data for C3 woody angiosperms in major biomes from compilation data in published literature (*g*_smax(lit)_) and this study (*g*_smax_). Bioclimatic zones are arranged from coldest to warmest

**Supplementary Table S10** Comparison of variance components (in percent, %) of stomatal conductance (*g_s_*) in the two habitats in this study

**Supplementary Table S11** Statistical description of photosynthetically active radiation (PAR, μmol m^-2^ s^-1^) in the environment of the understory-subcanopy habitat

**Supplementary Table S12** Post-hoc test by Wilcoxon rank-sum with Bonferroni correction, showing the *P* values of pairwise comparison between mean PAR of bioclimatic zones in the understory-subcanopy habitat

**Supplementary Table S13** Statistical description of photosynthetically active radiation (PAR, μmol m^-2^ s^-1^) in the environment of the open-canopy habitat

**Supplementary Table S14** Post-hoc test by Wilcoxon rank-sum with Bonferroni correction, showing the *P* values of pairwise comparison between mean PAR of bioclimatic zones in the open-canopy habitat

**Supplementary Table S15** Statistical description of vapor pressure deficit (VPD, kPa) in the environment of the understory-subcanopy habitat

**Supplementary Table S16** Post-hoc test by Wilcoxon rank-sum with Bonferroni correction, showing the *P* values of pairwise comparison between mean VPD of bioclimatic zones in the understory-subcanopy habitat

**Supplementary Table S17** Statistical description of vapor pressure deficit (VPD, kPa) in the environment of the open-canopy habitat

**Supplementary Table S18** Post-hoc test by Wilcoxon rank-sum with Bonferroni correction, showing the *P* values of pairwise comparison between mean VPD of bioclimatic zones in the open-canopy habitat

**Supplementary Table S19** Generalized Extreme Value (GEV) distribution parameters and lower and upper limits of the 95% CI *g*_smax(day)_

**Supplementary Data File (see separate Excel file)**

Maximum stomatal conductance (*g*_smax_) data derived from STraits (stomatal conductance, *g*_s_) dataset

| 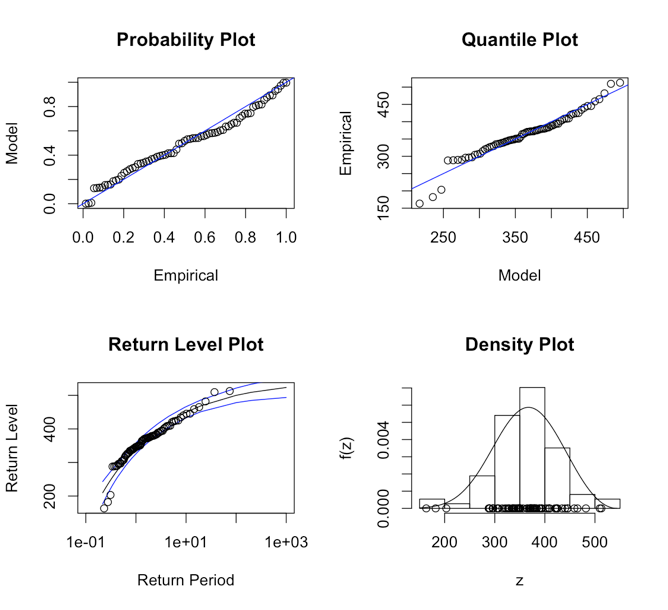  (a) | 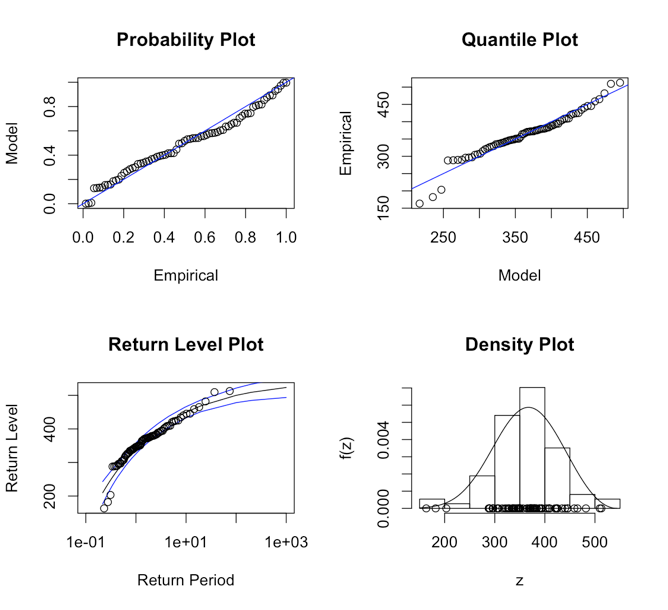  (b) |
| --- | --- |
| 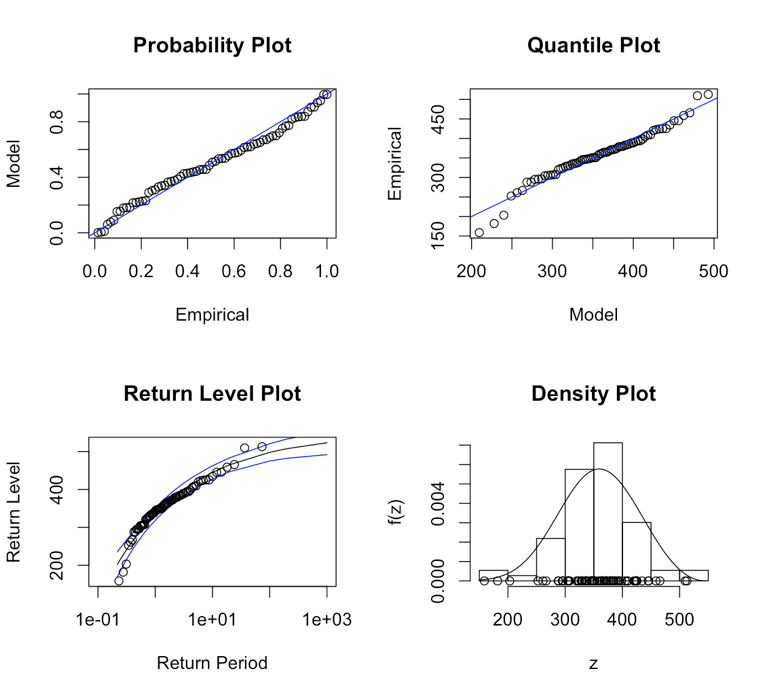  (c) | 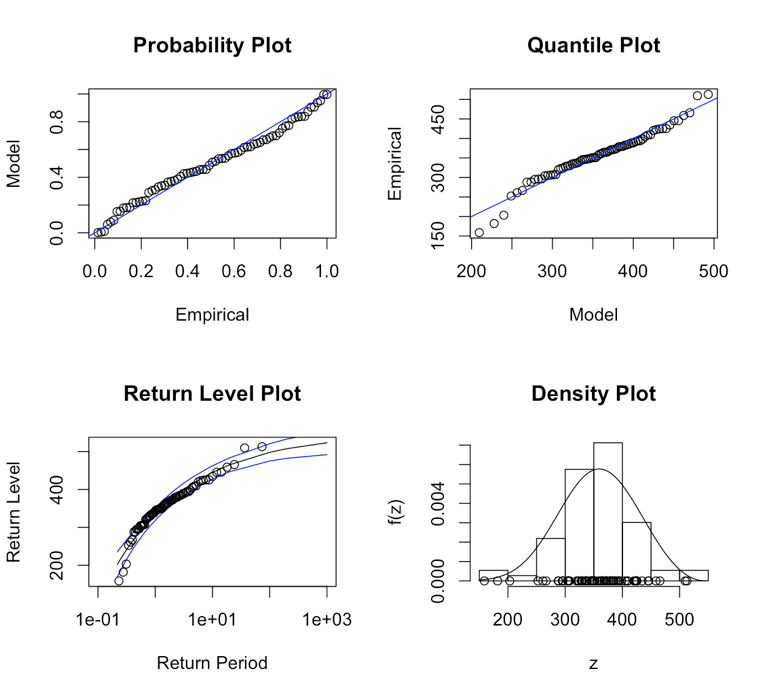  (d) |
| 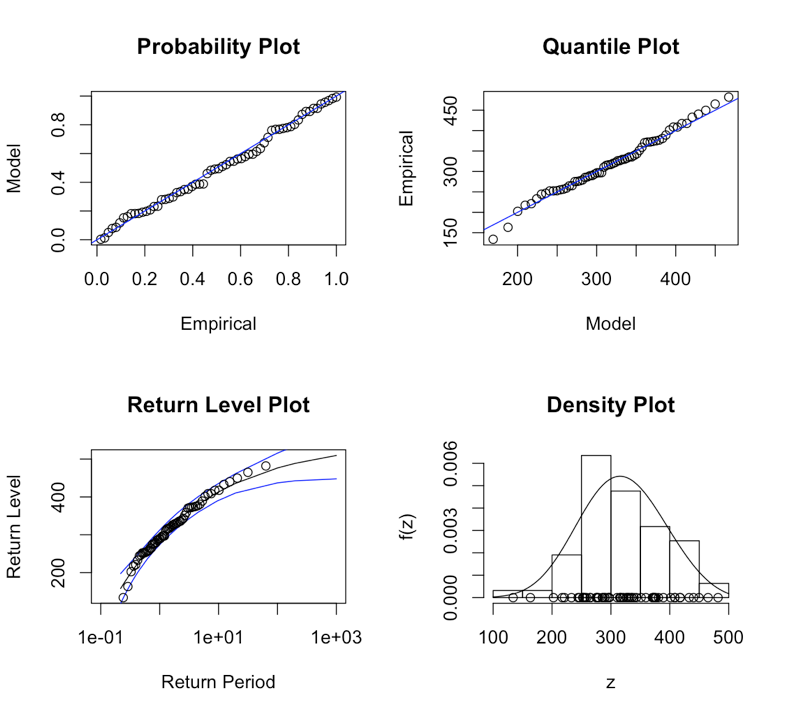  (e) | 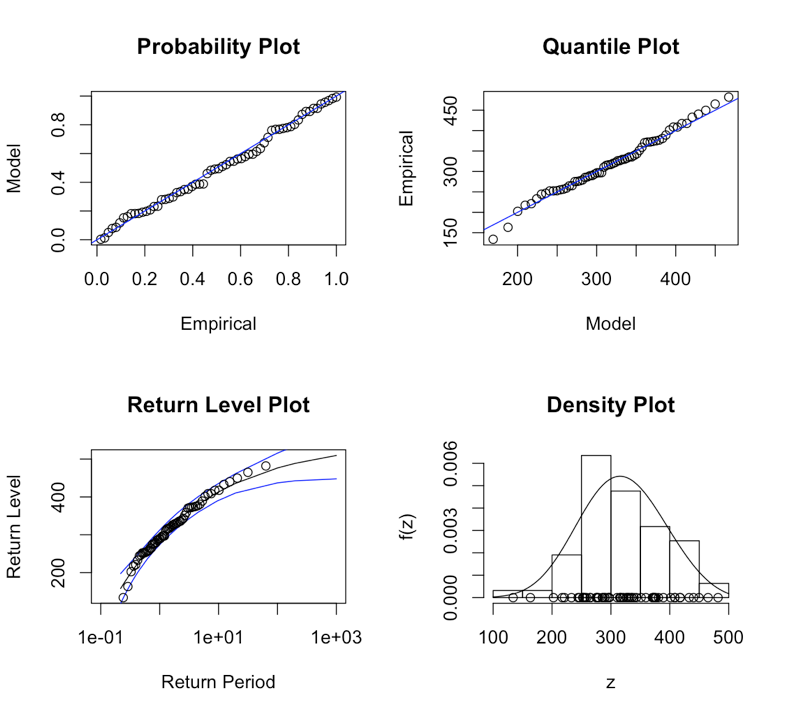  (f) |

**Figure S1**Diagnostic plots indicating goodness of fit of the Generalized Extreme Value (GEV) distribution to maximum stomatal conductance for a given day (*g*_smax(day)_). Panels (a), (c) and (e) show the fitted GEV density curve superimposed over a histogram of the actual data and panels (b), (d) and (f) are the respective quantile-quantile plots for the total dataset, (a) – (b), the open-canopy habitat, (c) – (d), and understory-subcanopy habitat, (d) – (e).

**Table S1** Post-hoc test by Tukey’s honest significant difference, showing the *P* values of pairwise comparison between mean *g*_smax_ of bioclimatic zones in understory-subcanopy habitat

| **Bioclimatic zone** | **BF** | **MED** | **TDF** | **TF** | **TRF** |
| --- | --- | --- | --- | --- | --- |
| **MED** | .57 | - | - | - | - |
| **TDF** | .76 | .99 | - | - | - |
| **TF** | .98 | .90 | .34 | - | - |
| **TRF** | .76 | .99 | .81 | .98 | - |
| **TSF(M)** | .45 | .99 | .99 | .79 | .97 |

**Table S2** Post-hoc test by Tukey’s honest significant difference, showing the *P* values of pairwise comparison between mean *g*_smax_ of bioclimatic zones in open-canopy habitat

| **Bioclimatic zone** | **BF** | **MED** | **TDF** | **TF** | **TRF** | **TSF(M)** |
| --- | --- | --- | --- | --- | --- | --- |
| **MED** | .55 | - | - | - | - | - |
| **TDF** | .01 | .40 | - | - | - | - |
| **TF** | .99 | .72 | .07 | - | - | - |
| **TRF** | .11 | .94 | .96 | .29 | - | - |
| **TSF(M)** | .93 | .99 | .97 | .93 | .99 | - |
| **SD** | < .001 | < .001 | < .001 | < .001 | < .001 | .03 |

**Table S3** Comparison of *g*_smax_ (mmol m^-2^ s^-1^) recorded at Jasper Ridge Biological Preserve for species common to both Maire et al. (2015) and STraits datasets, showing percentage difference in values

|  | **STraits** | **Maire et al. (2015)** | | | |
| --- | --- | --- | --- | --- | --- |
|  |  | Author | | Author | |
|  |  | Ackerly, D (2004) | | Field et al. (1983) | |
| Species | *g*_smax_ | *g*_smax_ | % difference | *g*_smax_ | % difference |
| *Arbutus menziesii* | 225 | 151 | 33 | 134 | 40 |
| *Baccharis pilularis* | 183 | 213 | -16 | - |  |
| *Ceanothus cuneatus* | 236 | 261 | -11 | - |  |
| *Ceanothus oliganthus* | 157 | 295 | -88 | - |  |
| *Eriodictyon californicum* | 359 | 352 | 2 | - |  |
| *Heteromeles arbutifolia* | 274 | 234 | 14 | 134 | 51 |
| *Holodiscus discolor* | 252 | 355 | -41 | - |  |
| *Mimulus aurantiacus* | 296 | 265 | 10 | - |  |
| *Prunus ilicifolia* | 283 | 134 | 53 | 163 | 42 |
| *Frangula californica* | 312 | 154 | 50 | 121 | 61 |
| *Rhamnus crocea* | 220 | 192 | 13 | - |  |
| *Umbellularia californica* | 68 | - |  | 148 | -116 |
| Average difference |  |  | 1.7 |  | 15.6 |

**Table S4** Comparison of the *g*_smax_/*g*_smax(lit)_ (mmol m^-2^ s^-1^) of common species from the same bioclimatic zones from the STraits dataset and Maire et al. (2015) dataset, showing percentage difference in values

| Bioclimatic zone | Species | *g*_smax(lit)_  (Maire et al. 2015) | *g*_smax_  (STraits) | Percentage difference (%) |
| --- | --- | --- | --- | --- |
| SD | *Baccharis salicifolia* | 424 | 304 | -28 |
| TDF | *Acer negundo* | 320 | 221 | -31 |
| TDF | *Acer rubrum* | 237 | 287 | 21 |
| TDF | *Carya ovata* | 316 | 210 | -34 |
| TDF | *Ilex verticillata* | 215 | 269 | 25 |
| TDF | *Kalmia latifolia* | 172 | 247 | 43 |
| TDF | *Liriodendron tulipifera* | 450 | 325 | -28 |
| TDF | *Populus tremuloides* | 431 | 275 | -36 |
| TDF | *Rhododendron maximum* | 136 | 232 | 70 |
| TDF | *Ulmus americana* | 454 | 311 | -31 |
| TDF | *Vaccinium corymbosum* | 145 | 251 | 73 |
| MED | *Arbutus menziesii* | 151 | 381 | 153 |
| MED | *Baccharis pilularis* | 213 | 250 | 18 |
| MED | *Ceanothus cuneatus* | 261 | 401 | 54 |
| MED | *Ceanothus oliganthus* | 295 | 262 | -11 |
| MED | *Eriodictyon californicum* | 352 | 364 | 3 |
| MED | *Frangula californica* | 154 | 510 | 230 |
| MED | *Heteromeles arbutifolia* | 234 | 379 | 62 |
| MED | *Holodiscus discolor* | 355 | 249 | -30 |
| MED | *Lepechinia calycina* | 198 | 127 | -36 |
| MED | *Mimulus aurantiacus* | 265 | 281 | 6 |
| MED | *Prunus ilicifolia* | 163 | 386 | 137 |
| MED | *Rhamnus crocea* | 192 | 221 | 15 |
| MED | *Toxicodendron diversilobum* | 213 | 332 | 56 |
| MED | *Umbellularia californica* | 148 | 65 | -56 |
| TSF | *Schefflera morototoni* | 1080 | 221 | -80 |
|  | Average difference |  |  | 22 |

**Table S5** Summary statistics of maximum stomatal conductance (*g*_smax,_ mmol m^-2^ s^-1^) for seven bioclimatic zones in this study for combined open-canopy and understory-subcanopy habitat

| Bioclimatic zone | *n* | spp. | Mean | SD | Median | Max | Min | 1st quartile | 3rd quartile | L(K-S)* |
| --- | --- | --- | --- | --- | --- | --- | --- | --- | --- | --- |
| Boreal forest | 65 | 30 | 286^A^ | 90 | 282 | 465 | 76 | 226 | 340 | 0.06 |
| Mediterranean | 71 | 44 | 269^A^ | 98 | 274 | 510 | 58 | 204 | 329 | 0.06 |
| Subtropical desert | 38 | 18 | 169^C^ | 99 | 144 | 423 | 11 | 95 | 245 | 0.12 |
| Temperate deciduous forest | 98 | 56 | 232^B^ | 80 | 232 | 434 | 73 | 181 | 281 | 0.06 |
| Temperate rainforest | 82 | 35 | 256^AB^ | 83 | 261 | 410 | 37 | 201 | 310 | 0.50 |
| Tropical rainforest | 52 | 34 | 267^A^ | 89 | 273 | 482 | 124 | 200 | 315 | 0.07 |
| Tropical seasonal forest (moist) | 24 | 22 | 234^ABC^ | 107 | 225 | 513 | 32 | 169 | 265 | 0.17 |

*n*, number of species-site values; SD, standard deviation; L(K-S), Lilliefors (Kolmogorov-Smirnov) test for departure from normality.

^A, B, C^ Values sharing the same letter are not significantly different by Tukey’s honest significant difference, *(P* < 0.05).

*All *P* values are > 0.05 for L(K-S) test, therefore all bioclimatic zones are normally distributed.

**Table S6** Post-hoc test by Tukey’s honest significant difference, showing the *P* values of pairwise comparison between mean *g*_smax_ of bioclimatic zones in combined understory-subcanopy and open-canopy habitat

| **Bioclimatic zone** | **BF** | **MED** | **TDF** | **TF** | **TRF** | **TSF(M)** |
| --- | --- | --- | --- | --- | --- | --- |
| **MED** | 0.91 | - | - | - | - | - |
| **TDF** | .003 | 0.10 | - | - | - | - |
| **TF** | .91 | 0.99 | 0.24 | - | - | - |
| **TRF** | .38 | 0.96 | 0.56 | 0.98 | - | - |
| **TSF(M)** | .18 | 0.63 | 0.99 | 0.73 | 0.94 | - |
| **SD** | < 0.001 | < 0.001 | 0.05 | < 0.001 | < 0.001 | 0.08 |

**Table S7** Summary statistics of maximum stomatal conductance (*g*_smax(lit),_ mmol m^-2^ s^-1^) for six bioclimatic zones in Maire et al. (2015). No data is available for woody angiosperm in the boreal bioclimatic zone.

| Bioclimatic zone | *n* | spp. | Mean | SD | Median | Max | Min | 1st quartile | 3rd quartile | L(K-S) |
| --- | --- | --- | --- | --- | --- | --- | --- | --- | --- | --- |
| Mediterranean | 165 | 135 | 223^B^ | 135 | 192 | 773 | 24 | 130 | 281 | 0.12* |
| Temperate deciduous forest | 124 | 103 | 201^B^ | 144 | 165 | 1050 | 37 | 104 | 249 | 0.13* |
| Tropical rainforest | 101 | 87 | 371^A^ | 351 | 300 | 2272 | 31 | 151 | 457 | 0.17* |
| Temperate rainforest | 23 | 17 | 242^AB^ | 83 | 215 | 422 | 131 | 186 | 301 | 0.15 |
| Tropical seasonal  forest (moist) | 137 | 123 | 313^A^ | 228 | 263 | 1307 | 65 | 140 | 389 | 0.15* |
| Subtropical desert | 17 | 17 | 247 ^AB^ | 164 | 176 | 631 | 55 | 124 | 400 | 0.20 |

No data were available for woody angiosperm in the boreal bioclimatic zone.

*n*, number of species-site values; SD, standard deviation; L(K-S), Lilliefors (Kolmogorov-Smirnov) test for departure from normality.

^A, B^ Values sharing the same letter are not significantly different by Tukey’s honest significant difference, *(P* < 0.05).

**P* < 0.05, therefore data is significantly different from normal distribution.

**Table S8** Post-hoc test by Tukey’s honest significant difference, showing the *P* values of pairwise comparison between mean *g*_smax_ of bioclimatic zones in Maire et al. (2015)

| **Bioclimatic zone** | **TF** | **TSF(M)** | **SD** | **TRF** | **MED** |
| --- | --- | --- | --- | --- | --- |
| **TSF(M)** | .29 | - | - | - | - |
| **SD** | .23 | .84 | - | - | - |
| **TRF** | .09 | .67 | .99 | - | - |
| **MED** | < .001 | .004 | .99 | .99 | - |
| **TDF** | < .001 | < .001 | .96 | .96 | .95 |

**Table S9** Comparison of maximum stomatal conductance data for C3 woody angiosperms in major biomes from compilation data in published literature (*g*_smax(lit)_) and this study (*g*_smax_). Bioclimatic zones are arranged from coldest to warmest.

| **Author** | **Biome/Bioclimatic zone** | ***g*_smax(lit)_ or *g*_smax_**  **(mmol m^-2^ s^-1^)** | **Species**  **(*n*)** | **Data points**  **(*n*)** | **Studies (*n*)** | **Year*** | **CO_2_ concentration (ppm)**** | **Method** |
| --- | --- | --- | --- | --- | --- | --- | --- | --- |
| Körner (1994) - biome descriptions according to Whittaker and Likens (1975) | Tundra | 253 | 14 | NA | c. 45 | 1971 to ~1988 | ~326 to ~352  (~26 ppm rise) | Porometer |
|  | Boreal (coniferous) | 234 | 26 | NA |  |  |  |  |
|  | Temperate deciduous forest | 190 | 22 | NA |  |  |  |  |
|  | Mediterranean | 219 | 41 | NA |  |  |  |  |
|  | Hot & cold desert shrublands | 200 | 9 | NA |  |  |  |  |
|  | Semi-arid subtropical/tropical shrub & woodland | 198 | 16 | NA |  |  |  |  |
|  | Seasonal tropical forest | ID | 4 | NA |  |  |  |  |
|  | Humid tropical forest | 249 | 17 | NA |  |  |  |  |
| Schulze et al. (1994) | Tundra | 242 | 12 | NA | NA | NA | NA | Porometer and IRGA |
|  | Temperate deciduous trees | 189 | 5 | NA |  |  |  |  |
|  | Temperate evergreen broadleaf | 209 | 5 | NA |  |  |  |  |
|  | Tropical deciduous forest | 271 | 47 | NA |  |  |  |  |
|  | Tropical rainforest | 205 | 210 | NA |  |  |  |  |
|  | Sclerophyllous shrubland | 197 | 42 | NA |  |  |  |  |
| Maire et al. (2015) -  biomes reclassified according to Whittaker (1974) | Tundra | 293 | 11 | 18 | 47 | 1977 to 2006 | ~334 to ~382  (~48 ppm rise) | IRGA |
|  | Temperate rainforest | 242 | 17 | 23 |  |  |  |  |
|  | Temperate deciduous forest | 201 | 103 | 124 |  |  |  |  |
|  | Mediterranean | 223 | 135 | 165 |  |  |  |  |
|  | Subtropical desert | 247 | 17 | 17 |  |  |  |  |
|  | Tropical seasonal forest (dry) and savanna | 315 | 53 | 60 |  |  |  |  |
|  | Tropical seasonal forest (moist) | 313 | 123 | 137 |  |  |  |  |
|  | Tropical rainforest | 371 | 87 | 101 |  |  |  |  |
| Murray et al. (this study) - biome classification based on Whittaker (1974) | Boreal forest | 286 | 30 | 65 | One study | 2012 to 2015 | ~394 to ~401  (~7 ppm rise) | Porometer corrected by IRGA |
|  | Temperate rainforest | 256 | 35 | 82 |  |  |  |  |
|  | Temperate deciduous forest | 232 | 56 | 98 |  |  |  |  |
|  | Mediterranean | 269 | 44 | 71 |  |  |  |  |
|  | Subtropical desert | 169 | 18 | 38 |  |  |  |  |
|  | Tropical seasonal forest (moist) | 234 | 22 | 24 |  |  |  |  |
|  | Tropical rainforest | 267 | 34 | 52 |  |  |  |  |

NA is not available

Studies (*n*) is the number of studies used to compile the dataset

*Year of data collection refers to the earliest and latest literature cited in the studies.

**Atmospheric CO_2_ concentration information corresponding to the collection year was obtained from a/the published instrumental dataset (1980 ‒ 2015) from Mauna Loa station (NOAA Earth System Research Laboratory, Global Monitoring Division (ftp://aftp.cmdl.noaa.gov/data/trace_gases/)).

Bioclimatic zones are arranged from coldest to warmest.

**Table S10** Comparison of variance components (in percent, %) of stomatal conductance (*g_s_*) in the two habitats in this study

| **Habitat** | **Species** | **Site** | **Bioclimatic zone** | **Residual** |
| --- | --- | --- | --- | --- |
| Open-canopy | 19 | 7 | 22 | 52 |
| Understory-subcanopy | 44 | 5 | 0 | 52 |

**Table S11** Statistical description of photosynthetically active radiation (PAR, μmol m^-2^ s^-1^) in the environment of the understory-subcanopy habitat

| **Bioclimatic zone** | ***n*** | **Mean** | **SD** | **Median** | **Max** | **Min** | **1st quartile** | **3rd quartile** | ***P* value of two-sample Wilcoxon rank-sum between two habitat groups** |
| --- | --- | --- | --- | --- | --- | --- | --- | --- | --- |
| Total | 2003 | 319 | 423 | 141 | 2642 | 4 | 54 | 376 | - |
| BF | 496 | 391 | 457 | 197 | 2642 | 16 | 89 | 518 | < .001 |
| MED | 201 | 376 | 464 | 187 | 1921 | 19 | 85 | 386 | < .001 |
| TDF | 317 | 309 | 377 | 158 | 1794 | 10 | 59 | 389 | < .001 |
| TF | 481 | 256 | 365 | 94 | 1890 | 5 | 36 | 315 | < .001 |
| TRF | 292 | 389 | 481 | 161 | 1835 | 7 | 58 | 505 | < .001 |
| TSF(M) | 216 | 163 | 333 | 52 | 2182 | 4 | 18 | 143 | < .001 |

The *n*-observation is the number of PAR measurements that were taken at the same time as stomatal conductance (*g*_s_) measurements. SD is standard deviation.

**Table S12** Post-hoc test by Wilcoxon rank-sum with Bonferroni correction, showing the *P* values of pairwise comparison between mean PAR of bioclimatic zones in the understory-subcanopy habitat

| **Bioclimatic zone** | **BF** | **MED** | **TDF** | **TF** | **TRF** |
| --- | --- | --- | --- | --- | --- |
| **MED** | 1 | - | - | - | - |
| **TDF** | .01 |  | - | - | - |
| **TF** | < .001 | < .001 | 0.001 | - | - |
| **TRF** | 0.82 | 1 | 1 | < .001 | - |
| **TSF(M)** | < .001 | < .001 | < .001 | < .001 | < .001 |

**Table S13** Statistical description of photosynthetically active radiation (PAR, μmol m^-2^ s^-1^) in the environment of the open-canopy habitat

| **Bioclimatic zone** | ***n*** | **Mean** | **SD** | **Median** | **Max** | **Min** | **1st quartile** | **3rd quartile** |
| --- | --- | --- | --- | --- | --- | --- | --- | --- |
| Total | 2041 | 896 | 605 | 795 | 2431 | 24 | 342 | 1442 |
| Total excl. SD | 1589 | 913 | 620 | 878 | 2431 | 24 | 317 | 1476 |
| BF | 292 | 658 | 594 | 381 | 2431 | 37 | 194 | 1090 |
| MED | 507 | 1182 | 625 | 1351 | 2155 | 27 | 595 | 1711 |
| SD | 452 | 840 | 548 | 638 | 2324 | 63 | 420 | 1240 |
| TDF | 271 | 890 | 604 | 832 | 2258 | 37 | 323 | 1375 |
| TF | 87 | 840 | 558 | 724 | 2161 | 61 | 366 | 1222 |
| TRF | 365 | 868 | 517 | 866 | 2069 | 24 | 398 | 1333 |
| TSF | 67 | 415 | 452 | 195 | 2078 | 47 | 141 | 479 |

The *n*-observation is the number of PAR measurements that were taken at the same time as stomatal conductance (*g*_s_) measurements. SD is standard deviation.

**Table S14** Post-hoc test by Wilcoxon rank-sum with Bonferroni correction, showing the *P* values of pairwise comparison between mean PAR of bioclimatic zones in the open-canopy habitat

| **Bioclimatic zone** | **BF** | **MED** | **TDF** | **TF** | **TRF** | **TSF(M)** |
| --- | --- | --- | --- | --- | --- | --- |
| **MED** | < .001 | - | - | - | - | - |
| **TDF** | < .001 | < .001 | - | - | - | - |
| **TF** | .019 | < .001 | 1.00 | - | - | - |
| **TRF** | < .001 | < .001 | 1.00 | 1.00 | - | - |
| **TSF(M)** | .009 | < .001 | < .001 | < .001 | < .001 | - |
| **SD** | < .001 | < .001 | 1.00 | 1.00 | 1.00 | < .001 |

**Table S15** Statistical description of vapor pressure deficit (VPD, kPa) in the environment of the understory-subcanopy habitat

| **Bioclimatic zone** | ***n*** | **Mean** | **SD** | **Median** | **Max** | **Min** | **1st quartile** | **3rd quartile** | ***P* value of two-sample Wilcoxon rank-sum between two habitat groups** |
| --- | --- | --- | --- | --- | --- | --- | --- | --- | --- |
| Total | 2003 | 0.82 | 0.49 | 0.73 | 3.09 | 0.01 | 0.48 | 1.06 | - |
| BF | 496 | 0.78 | 0.41 | 0.69 | 3.02 | 0.20 | 0.49 | 1.00 | < .001 |
| MED | 201 | 0.98 | 0.65 | 0.70 | 2.90 | 0.15 | 0.43 | 1.43 | < .001 |
| TDF | 317 | 0.97 | 0.65 | 0.74 | 3.09 | 0.05 | 0.51 | 1.39 | < .001 |
| TF | 481 | 0.82 | 0.33 | 0.78 | 2.47 | 0.08 | 0.58 | 0.99 | < .001 |
| TRF | 292 | 0.50 | 0.38 | 0.40 | 2.16 | 0.01 | 0.20 | 0.73 | < .001 |
| TSF(M) | 216 | 1.00 | 0.38 | 0.96 | 2.49 | 0.28 | 0.72 | 1.25 | 0.903 |

The *n*-observation is the number of VPD measurements that were taken at the same time as stomatal conductance (*g*_s_) measurements. SD is standard deviation.

**Table S16** Post-hoc test by Wilcoxon rank-sum with Bonferroni correction, showing the *P* values of pairwise comparison between mean VPD of bioclimatic zones in the understory-subcanopy habitat

| **Bioclimatic zone** | **BF** | **MED** | **TDF** | **TF** | **TRF** |
| --- | --- | --- | --- | --- | --- |
| **MED** | .94 | - | - | - | - |
| **TDF** | .07 | 1.00 | - | - | - |
| **TF** | .03 | 1.00 | 1.00 | - | - |
| **TRF** | < .001 | < .001 | < .001 | < .001 | - |
| **TSF(M)** | < .001 | .26 | .03 | < .001 | < .001 |

**Table S17** Statistical description of vapor pressure deficit (VPD, kPa) in the environment of the open-canopy habitat

| **Bioclimatic zone** | ***n*** | **Mean** | **SD** | **Median** | **Max** | **Min** | **1st quartile** | **3rd quartile** |
| --- | --- | --- | --- | --- | --- | --- | --- | --- |
| Total | 2041 | 1.38 | 0.93 | 1.15 | 5.68 | 0.03 | 0.63 | 1.89 |
| Total excl. SD | 1589 | 1.07 | 0.68 | 0.92 | 4.25 | 0.03 | 0.54 | 1.89 |
| BF | 292 | 0.92 | 0.42 | 0.90 | 2.46 | 0.26 | 0.57 | 1.13 |
| MED | 507 | 1.28 | 0.76 | 1.15 | 4.25 | 0.11 | 0.66 | 1.77 |
| SD | 452 | 2.47 | 0.89 | 2.41 | 5.68 | 0.65 | 1.76 | 2.99 |
| TDF | 271 | 1.40 | 0.83 | 1.42 | 3.70 | 0.03 | 0.59 | 1.98 |
| TF | 87 | 1.07 | 0.48 | 1.08 | 2.17 | 0.19 | 0.66 | 1.47 |
| TRF | 365 | 0.66 | 0.38 | 0.59 | 2.36 | 0.04 | 0.37 | 0.88 |
| TSF(M) | 67 | 1.03 | 0.49 | 0.94 | 2.36 | 0.24 | 0.63 | 1.40 |

The *n*-observation is the number of VPD measurements that were taken at the same time as the stomatal conductance (*g*_s_) measurements. SD is standard deviation.

**Table S18** Post-hoc test by Wilcoxon rank-sum with Bonferroni correction, showing the *P* values of pairwise comparison between mean VPD of bioclimatic zones in the open-canopy habitat

| **Bioclimatic zone** | **BF** | **MED** | **TDF** | **TF** | **TRF** | **TSF(M)** |
| --- | --- | --- | --- | --- | --- | --- |
| **MED** | < .001 | - | - | - | - | - |
| **TDF** | < .001 | 0.92 | - | - | - | - |
| **TF** | .11 | 1.00 | .04 | - | - | - |
| **TRF** | < .001 | < .001 | < .001 | < .001 | - | - |
| **TSF(M)** | 1.00 | 0.91 | 0.052 | 1.00 | < .001 | - |
| **SD** | < .001 | < .001 | < .001 | < .001 | < .001 | < .001 |

**Table S19** Generalized Extreme Value (GEV) distribution parameters and lower and upper limits of the 95% CI *g*_smax(day)_

|  | Modelled GEV parameters | | |  |  |
| --- | --- | --- | --- | --- | --- |
| Dataset | Location | Scale | Shape | Lower and upper limits of 95% CI *g*_smax(day)_  (mmol m^-2^ s^-1^) | Percentage of *g*_smax_ data points >50% the lower limit of 95% CI *g*_smax(day)_ |
| Entire dataset | 342.03  (325.69, 358.37) | 66.61  (55.76, 77.47) | -0.33  (-0.43, -0.23) | 233, 484 | 85% |
| Open-canopy | 335.01  (318.35, 351.68) | 67.49  (56.42, 78.56) | -0.32  (-0.42, -0.22) | 226, 481 | 92% |
| Understory-subcanopy | 294.15  (274.78, 313.52) | 71.23  (57.7, 84.75) | -0.29  (-0.44, -0.13) | 181, 456 | 89% |

Values in parentheses are the 95% CI.
